# Photoelectrochemical nitrate reduction to ammonia on CuFeO_2_ photocathodes modified with metallic cocatalysts

**DOI:** 10.1039/d6ra05930a

**Published:** 2026-09-21

**Authors:** Mohamed El Idrissi, Anastasiia Kotova, Ying Kong, Muhammad Adib Abdillah Mahbub, Mohammed Abd-Lefdil, Lahoucine Atourki, Bastian Timo Mei

**Affiliations:** a Laboratory of Industrial Chemistry, Ruhr-University Bochum Universitätsstraße 150 Bochum 44801 Germany bastian.mei@rub.de; b MANAPSE Lab, Faculty of Sciences Avenue Ibn Batouta Rabat P.B. 1014 Morocco; c Analytical Chemistry – Center for Electrochemical Sciences (CES), Ruhr-University Bochum Universitätsstraße 150 Bochum 44801 Germany

## Abstract

Photoelectrochemical nitrate reduction (p-NO_3_RR) is a sustainable route to green ammonia that simultaneously removes excess nitrate from wastewater thereby preventing ecosystem damage. In this work, CuFeO_2_, a p-type delafossite semiconductor, is investigated as a photocathode for p-NO_3_RR. Unmodified CuFeO_2_ is demonstrated to be unable to facilitate liquid p-NO_3_RR product formation, however, cocatalyst screening (Pt, Au, and Co) revealed that for all cocatalyst-modified CuFeO_2_ photoelectrodes, ammonia formation is observed, with Pt being the superior cocatalytic material for selective nitrate reduction. Optimisation of the Pt hydrothermal immobilization procedure resulted in the preparation of CuFeO_2_/Pt photocathodes, achieving a faradaic efficiency (FE) of 92% toward ammonia at a low potential of 0.63 V *vs.* RHE, with improved short-term stability. Surface characterisation of the photocathodes indicated that the enhanced stability of the metal-modified CuFeO_2_ photocathodes during p-NO_3_RR compared to unmodified CuFeO_2_ is attributable to the formation of a Cu^2+^-containing surface layer obtained during hydrothermal processing under strongly alkaline conditions. This layer may impede reduction of Cu^+^ to metallic copper, a frequently reported degradation mechanism for delafossite photoelectrodes. While further improvements in long-term stability are still required, these findings clearly demonstrate the viability of CuFeO_2_-based photocathodes for selective p-NO_3_RR, *i.e.* for sustainable ammonia synthesis from nitrate waste streams, and highlight the material's potential for solar-to-chemical energy conversion.

## Abstract 2

Modification and optimisation of CuFeO_2_ with Pt resulted in photoelectrochemical nitrate reduction with a FE of 92%.

## Graphical abstract

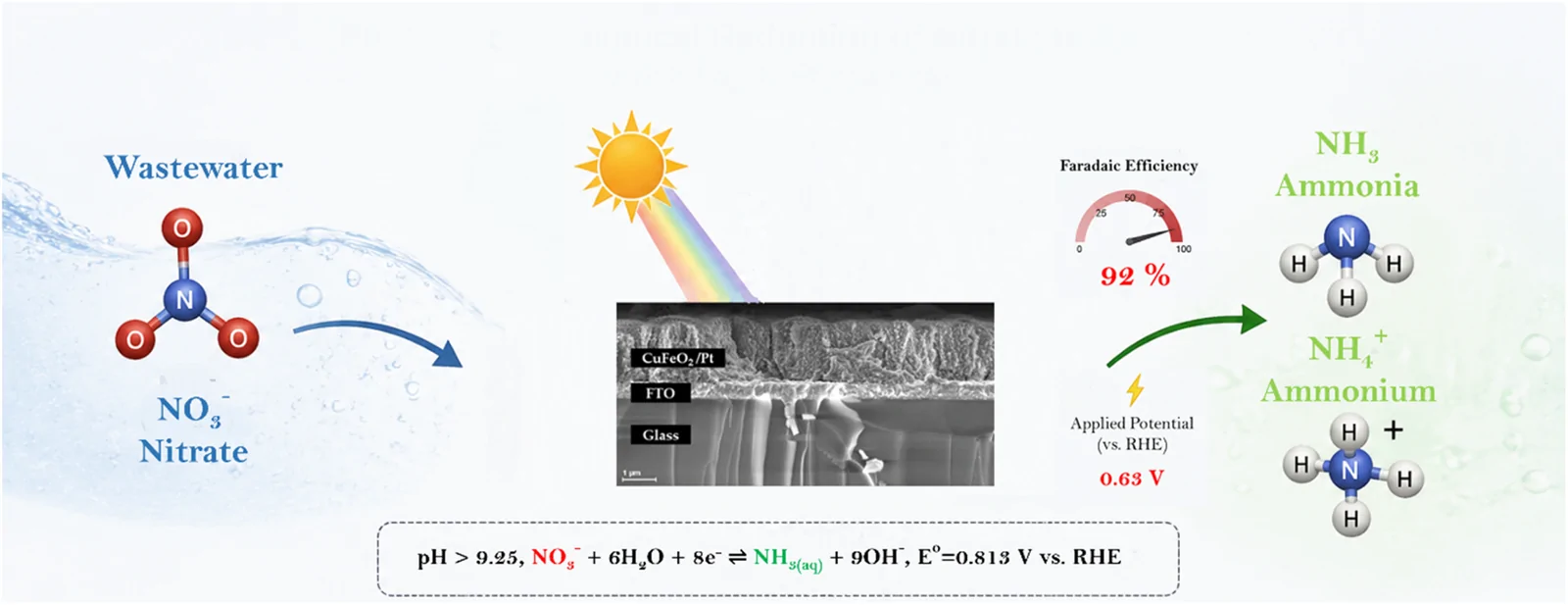

## Introduction

Ammonia (NH_3_) is emerging as a promising energy carrier due to its high energy density (3 kW h kg^−1^), high hydrogen content (17.7 wt%),^1–3^ and ease of storage and transportation.^4^ These attributes make ammonia a resilient and sustainable energy solution for future sustainable energy scenarios.^5^ Beyond its role as an energy carrier, NH_3_ is a critical component in nitrogen fertilizers and the chemical industry,^6–9^ where it plays an essential role in sustaining agricultural productivity and supporting industrial processes.

Currently, the industrial production of NH_3_ relies predominantly on the Haber-Bosch process, which accounts for over 90% of the global ammonia production.^10^ However, this method operates under high-temperature and high-pressure conditions, with the required hydrogen production leading to significant fossil fuel consumption and greenhouse gas emissions.^11–14^ Annually, the ammonia industry consumes 2–3% of the world's natural gas production (equivalent to 1–2% of the global energy supply) and contributes substantially to global carbon emissions.^15–17^ While the increasing global population demands higher crop yields and thus greater ammonia production, its environmental impact necessitates the development of alternative, sustainable production methods.^18,19^

Ammonia synthesis under mild conditions powered by renewable energy sources has emerged as a promising solution, particularly for decentralized production scenarios.^20–22^ Among these, the electrocatalytic nitrogen reduction reaction (e-N_2_RR) has gained significant attention due to its ability to convert nitrogen (N_2_) to ammonia using water as the hydrogen source and renewable electricity as the energy input.^23–26^ However, e-N_2_RR faces major challenges, including the low solubility of N_2_ in aqueous solutions (1.17 × 10^−5^ M at 25 °C and 1 atm), the high dissociation energy of the N≡N triple bond (941 kJ mol^−1^), and the competitive hydrogen evolution reaction (HER). These factors severely limit the faradaic efficiency (FE) and ammonia yield, which impedes its scalability and industrial application.^27–31^ As an alternative reactant to atmospheric nitrogen, nitrate (NO_3_^−^) is receiving increasing attention as a water-soluble nitrogen source for catalytic ammonia synthesis. Nitrate offers several advantages over N_2_, including a relatively low average N–O bond dissociation energy (204 kJ mol^−1^), reflecting the resonance delocalized nature of its bonding.^32–35^ Nitrate reduction not only enables ammonia production but also provides a means to mitigate environmental pollution by removing harmful nitrate ions from wastewater and other sources.^36–38^ Excessive nitrate levels in surface and groundwater, often resulting from industrial discharge, agricultural runoff, and animal waste, pose significant environmental and health risks,^39–41^ including eutrophication and water contamination. Therefore, nitrate reduction provides a distinct advantage as a “waste-to-energy” technology, addressing environmental pollution and enabling sustainable decentralized NH_3_ production concurrently.

Electrocatalytic nitrate reduction (e-NO_3_RR) to ammonia is frequently investigated using noble (*e.g.*, Ru,^42^ Ir,^43^ Pd,^44^ Pt,^45^ Au^46^) and transition metal (*e.g.*, Cu,^47,48^ Ti,^49,50^ Ni,^51^ Co,^52^ Fe^37^) catalysts. Recent advancements in copper–iron composite materials have provided valuable insights into the mechanisms of e-NO_3_RR. For instance, a dual-site Cu–Fe catalyst has been shown to stabilize nitrite intermediates and promote their selective hydrogenation to ammonia.^53^ However, the process is kinetically demanding (eight electron transfer reaction). At pH 0–7, the overall reaction is NO_3_^−^ + 10H^+^ + 8e^−^ ⇌ NH_4_^+^ + 3H_2_O, *E*° = 0.88 V *vs.* RHE. Between pH 7–9.25, the reaction proceeds as NO_3_^−^ + 7H_2_O + 8e^−^ ⇌ NH_4_^+^ + 10OH^−^, *E*° = 0.88 V *vs.* RHE and at pH levels >9.25, the pathway shifts to NO_3_^−^ + 6H_2_O + 8e^−^ ⇌ NH_3(aq)_ + 9OH^−^, *E*° = 0.813 V *vs.* RHE.^54^ Importantly nitrate reduction faces competition from undesirable intermediates and byproduct formation including hydrogen (H_2_), nitrite (NO_2_^−^), nitric oxide (NO), dinitrogen (N_2_), and hydroxylamine (NH_2_OH).^55^ Moreover despite significant advancements in the field, its practical applicability is still limited by high overpotentials, especially in neutral environments where nitrate-containing wastewater is prevalent, rendering the process rather energy inefficient.^56–59^ Electrolyte pH is therefore a critical parameter in determining e-NO_3_RR selectivity. In near-neutral or acidic media, nitrate reduction often favours nitrite or hydroxylamine intermediates, whereas strongly alkaline conditions bias the reaction pathway toward ammonia formation by stabilizing proton-coupled electron transfer steps.^60^

Photoelectrochemical (PEC) nitrate reduction (p-NO_3_RR) is another less frequently explored approach for nitrate utilization.^61^ PEC systems combine the advantages of electrocatalysis and photocatalysis, utilizing solar energy to generate an internal photovoltage. As such nitrate reduction in p-NO_3_RR is generally observed at lower applied potentials than in e-NO_3_RR.^62,63^ Given that the reduction potential of nitrate is more positive than that of water, PEC water-splitting materials are also considered suitable for p-NO_3_RR.^64^ Copper-based photoelectrodes have shown considerable promise for p-NO_3_RR. For example, Cu_2_O photocathodes have demonstrated enhanced photostability and selectivity towards NH_3_. Yet only moderate faradaic efficiencies (FE_NH_3__) of 20% under visible light illumination were achieved.^65^ Modifications such as the incorporation of dual-function catalysts and *in situ* transformations of the photoelectrode surface (*e.g.*, CuO to Cu/Cu_2_O) have been shown to further improved selectivity. Still, Cu-based photoelectrodes often favour nitrite formation (presumably also as a result of the neutral electrolyte), limiting their ammonia selectivity.^66^

CuFeO_2_ crystallizes in either the rhombohedral (3R) or hexagonal (2H) polytypes, with space groups *R*3̄*m* and *P*6_3_/*mmc*, respectively. The valence and conduction band edges in CuFeO_2_ delafossites are predominantly composed of Cu 3d and Fe d/s orbitals, which provide dual-site redox functionality.^67^ With a band gap of 1.55 eV, an absorption coefficient up to *α* = 10^7^ m^−1^, and a flat-band potential located around 1 V *vs.* RHE, CuFeO_2_ combines visible-light absorption with sufficient photovoltage to drive multi-electron reduction reactions.^68^ Importantly, its layered delafossite structure confers enhanced stability in neutral and alkaline aqueous environments compared to Cu_2_O, where self-reduction to metallic Cu is a frequent degradation pathway.^69^ These structure–property features suggest that CuFeO_2_ could outperform Cu_2_O and related p-type semiconductors in p-NO_3_RR by leveraging Cu–Fe synergy for nitrate adsorption and hydrogenation, while maintaining photovoltage and suppressing instability. In recent studies, we demonstrated that CuFeO_2_, a layered delafossite p-type semiconductor, generates sufficient photovoltage to drive water splitting in alkaline electrolyte (photocurrent ≈ 1.7 mA cm^−2^ and FE ≈ 40% for HER).^70^ Considering that dual-site Cu–Fe were reported to promote ammonia formation^53,71^ a similar synergistic mechanism might be envisioned for CuFeO_2_ photocathodes. Nevertheless, unmodified CuFeO_2_ was found to be inherently unstable,^68^ underscoring the necessity for surface modification to enhance its stability.

This work aimed to achieve efficient NO_3_RR by leveraging the advantages offered by photoelectrochemical systems and utilizing the recently developed CuFeO_2_ photocathode for p-NO_3_RR. Here, Co, Au, and Pt were selected as potential cocatalysts to enhance the p-NO_3_RR performance of CuFeO_2_ due to their distinct electronic structures, which are believed to result in different NO_3_RR activities. The comprehensive cocatalyst screening revealed that modification of CuFeO_2_ with platinum cocatalyst facilitates nitrate reduction surpassing the selectivity to ammonia (FE_NH_3__) obtained with Co and Au modification. For optimized CuFeO_2_/Pt photocathodes, a faradaic efficiency for ammonia production of 92% at an applied potential of 0.63 V *vs.* RHE is obtained in alkaline electrolyte. Other liquid phase byproducts such as nitrite or hydroxylamine were not detected, highlighting the superior selectivity of the optimized CuFeO_2_/Pt photocathode at modest stability. Importantly, blank experiments in nitrate-free electrolyte showed no detectable ammonia signal (below the detection limit of 1.95 µM), whereas the optimized CuFeO_2_/Pt photocathode produced 27.8 µM NH_4_^+^ within 1 h, 14× above the detection limit. Moreover, this is well above false-positive ammonia detection^72^ with typical contamination levels of 1–10 µM.^73^ Beyond contamination, a central challenge was the instability of CuFeO_2_ photoelectrodes, particularly caused by the reduction of Cu^+^ to metallic Cu under cathodic bias strongly influencing durability. In this context, cocatalyst screening was used not only to enhance NO_3_RR activity but also to test whether surface modification could suppress the Cu^+^ → Cu^0^ reduction pathway and thereby improve operational robustness. The improved short-term stability of the photoelectrode was assigned to a Cu^+^ to Cu^2+^ oxidation due to the hydrothermal deposition procedure, post-catalytic characterization revealed a partial reduction of Fe^3+^ to Fe^2+^ being indicative of a potential detrimental parasitic side reaction. Nonetheless, the present study demonstrates the efficacy of p-NO_3_RR and identifies CuFeO_2_-based photocathodes as promising candidates for selective and efficient PEC nitrate reduction, thereby addressing the lack of prior work on delafossite materials in this field and providing a new avenue for sustainable ammonia production.

## Experimental section

### Reagents and materials

Copper(ii) sulphate pentahydrate (CuSO_4_·5H_2_O, 99.5%), iron(ii) sulphate heptahydrate (FeSO_4_·7H_2_O, 99.0%), urea (99.0%), HPLC-grade water, fluorine-doped tin oxide (FTO) glass slides (sheet resistance ≈ 7 Ω sq^−1^), chloroplatinic acid (H_2_PtCl_6_), gold(iii) chloride (HAuCl_4_), cobalt(ii) nitrate (Co(NO_3_)_2_·6H_2_O), sodium hydroxide (1.0 M NaOH), sodium salicylate (salicylic acid salt), sodium citrate, sodium hypochlorite (NaClO), sodium nitroprusside (C_5_FeN_6_Na_2_O), ammonium chloride (NH_4_Cl), sulfanilamide, hydrochloric acid (2.0 M HCl), *N*-(1-naphthyl)ethylenediamine dihydrochloride, potassium nitrite (KNO_2_), hydroxylamine (NH_2_OH) hydroxylamine hydrochloride, 8-quinolinol, sodium carbonate solution, absolute ethanol (all reagents purchased from Sigma-Aldrich).

### Sample synthesis

**Scheme 1:**
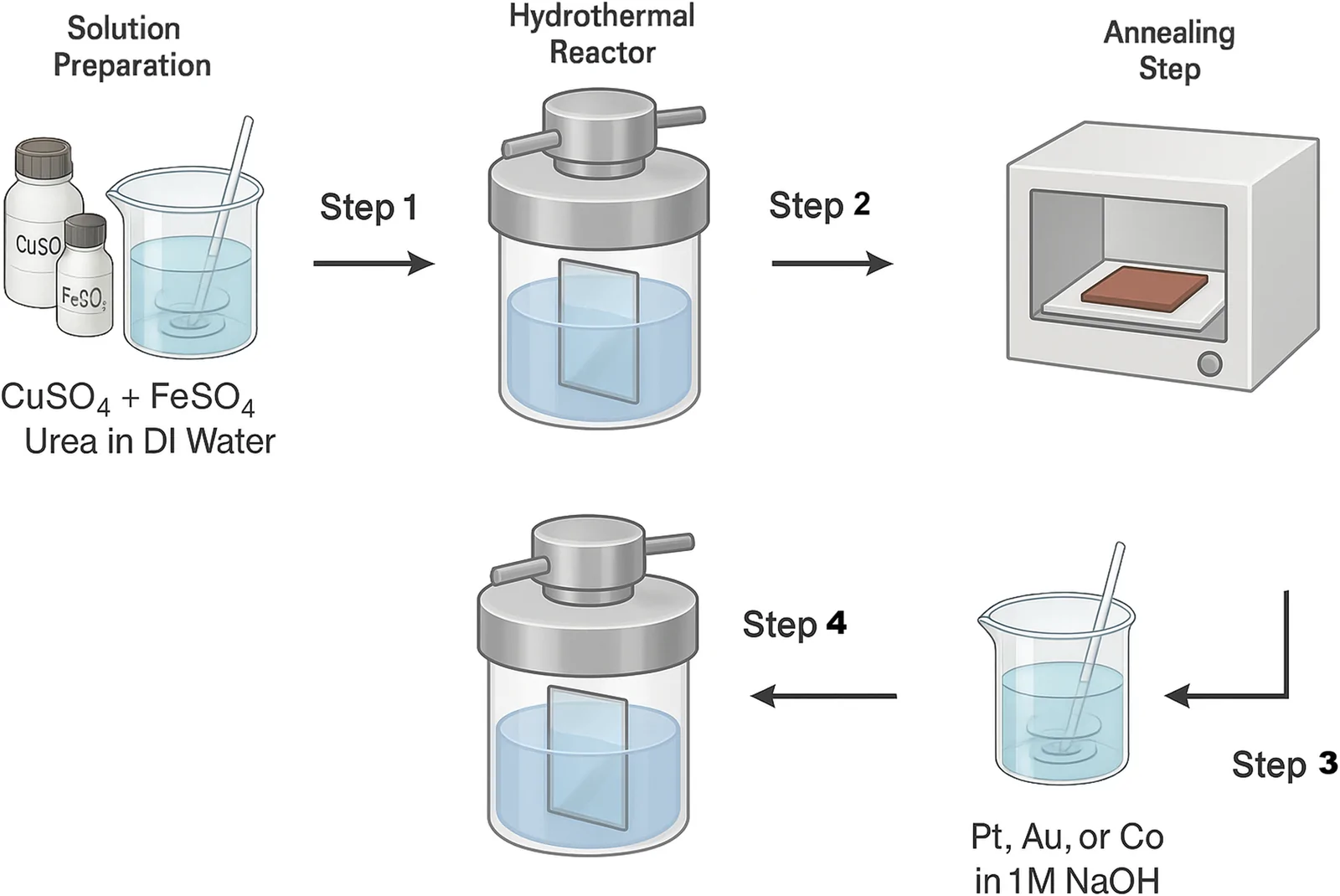
Schematic representation of the hydrothermal synthesis process for CuFeO_2_ on FTO glass substrates (steps 1 and 2 according to ref. 69) and subsequent deposition of noble and transition metals (Co, Au, or Pt) using a second hydrothermal step (steps 3 and 4).

Delafossite CuFeO_2_ photoelectrodes were prepared on fluorine-doped tin oxide (FTO) glass substrates using an optimized direct hydrothermal film growth procedure.^70^ In short, phase-pure CuFeO_2_ thin films were prepared by an one-step hydrothermal method at 110 °C (1 h) from aqueous solutions of copper(ii) sulphate (CuSO_4_), iron(ii) sulphate (FeSO_4_) and urea and subsequent annealing at 650 °C for 1 h (step 1 & 2, see Scheme 1). The as-prepared CuFeO_2_ were immersed in 1 M NaOH solution with the corresponding transition metals (Co, Au, Pt), followed by hydrothermal deposition (110 °C for 1 h), (step 3 & 4, see Scheme 1). A full description of the experimental details of the hydrothermal procedure is provided in the SI, together with the calculation method used to derive cocatalyst loadings from precursor volume and concentration.

### Materials characterization

Surface morphology and film integrity of the prepared cocatalyst modified CuFeO_2_ thin films were characterized by scanning electron microscopy (SEM). The elemental composition was probed by energy-dispersive X-ray spectroscopy (EDX). Imaging and EDX mapping carried out on a Zeiss Merlin Gemini II field-emission SEM equipped with a Schottky field emitter. The microscope was operated at an accelerating voltage of 5–15 kV depending on the imaging technique or the analytical requirement. To prevent charging and to ensure reliable imaging and quantification, a thin carbon coating applied with a Cressington 208 carbon coater prior to analysis modified the photoelectrodes.

Optical properties of the bare and modified CuFeO_2_ thin-film photocathodes (Co, Au, Pt modifications) were measured by UV–Vis spectroscopy. UV-Vis spectra recorded in the wavelength range from 200 to 900 nm using a PerkinElmer Lambda 650 spectrophotometer equipped with a PKI 100 mm integrating sphere accessory. All spectra acquired at room temperature and baseline corrections applied using an FTO substrate to remove substrate contributions.

X-ray photoelectron spectroscopy (XPS) was used to probe surface composition and oxidation states. Measurements were performed on a Kratos Axis Supra spectrometer with a monochromatic Al Kα X-ray source (*hν* = 1486.68 eV) under ultra-high vacuum (base pressure < 10^−9^ mbar). Survey scans and high-resolution spectra were collected for the Cu 2p, Fe 2p, O 1s, Co 2p, Pt 4f and Au 4f regions. Binding energies were referenced to the C 1s peak at 284.8 eV. Spectral processing and peak fitting carried out in CasaXPS using a Shirley background subtraction and Gaussian–Lorentzian peak shapes.

### (Photo)electrochemical measurements

Photoelectrochemical (PEC) experiments were performed in a three-electrode configuration. The working electrode was the FTO substrate uniformly coated with CuFeO_2_ thin films (nominal substrate size 1.0 × 2.0 cm). A 1.0 × 1.0 cm section of the CuFeO_2_ film was removed to expose the underlying FTO for electrical contact. Pt (≈2 × 2 cm) served as the counter electrode and a low-profile Ag/AgCl (4 M KCl gel, Pine Research) electrode was used as the reference. All current densities reported are normalized to the defined active area (1.0 cm^2^). Measurements were carried out in aqueous electrolytes containing 1.0 M NaOH (pH ≈ 13.2) with added KNO_3_ at the concentrations specified (typical nitrate concentration: 100 mM KNO_3_). Prior to each experiment the electrolyte was purged with high-purity N_2_ for 30 min to remove dissolved O_2_.

The photoelectrochemical cell consisted of a custom H-cell, with two compartments separated by an anion exchange membrane (FM-FAA-3PK-130, Fumasep, QuinTech). The working electrode compartment was equipped with a quartz optical window to allow front-side illumination. The membrane (130 µm thickness, polyketone reinforcement) provides mechanical stability and prevents crossover of dissolved species between the counter and working electrode compartments.

Illumination of the photoelectrode was provided by a MAX-303 xenon lamp (300 W, Asahi Spectra) equipped with a collimator lens and filter wheel. The light was directed from the front side of the electrode (through the electrolyte). The irradiance at the sample surface was maintained at 1 sun (100 mW cm^−2^) verified by a certified reference photodiode (Thorlabs). Linear sweep voltammetry (LSV) and transient photocurrent measurements were recorded under chopped illumination with a 50% duty cycle (for example, 5 s light-on/5 s light-off unless otherwise stated).

LSV was recorded from 0 to −0.65 V *vs.* Ag/AgCl (equivalent to +1.00 to +0.35 V *vs.* RHE under the conditions specified) at a scan rate of 5 mV s^−1^. Potentials are reported *versus* the reversible hydrogen electrode (RHE) using the following conversion:

1 *E*_RHE_ = *E*_Ag/AgCl_ + 0.197 V + 0.0591pH

The photoelectrode stability was determined by chronoamperometry (CA) using continuous illumination unless otherwise noted.

Incident photon-to-current efficiency (IPCE) in the visible (400–900 nm) was measured using a monochromatic light source (photoelectrochemical measurement system, Instytut Fotonowy, ver. 5.3) using the same three-electrode configuration and electrolyte (100 mM KNO_3_ in 1.0 M NaOH, N_2_-purged) specified above. The incident light intensity at each wavelength was measured and calibrated with a photodiode placed at the rear of the PEC cell. The exposed active area for these measurements was (0.9/2) 2π cm^2^.

Electrochemical impedance spectroscopy (EIS) was used to evaluate the impedance response of the cocatalyst modified CuFeO_2_ photocathodes under p-NO_3_RR. Measurements were performed at 0.63 V *vs.* RHE in 100 mM KNO_3_/1.0 M NaOH in the frequency range from 100 kHz to 10 Hz (using an AC perturbation amplitude of 10 mV) under illuminated conditions (1 sun illumination). The distribution of relaxation times (DRT) analysis^74^ was employed to deconvolute the impedance spectra and to guide the selection of an appropriate equivalent-circuit model. Based on that, the EIS data were fitted using Impedance.py^75^ with two-time-constant equivalent circuit model:^70^

2 *Z*(*ω*) = *R*_0_ + (*R*_1_‖CPE_1_) + (*R*_2_‖CPE_2_)

where *R*_0_ was assigned to the uncompensated solution resistance. Two *R*_*x*_‖CPE_*x*_ (*x* = 1, 2) elements were used to describe the faster and slower relaxation contributions, respectively, identified from the DRT analysis, and are not uniquely assigned to elementary processes. The impedance of the constant phase element was defined as *Z*(CPE) = 1/(*jω*)^*α*^*Q*, where *Q* is the CPE coefficient and *α* describes the deviation from ideal capacitive behavior. The fitted resistances and CPE parameters obtained from the equivalent-circuit analysis are summarized in Table S1.

*Operando* Raman spectrophotoelectrochemical measurements were performed using a LabRAM HR Raman microscopy system (Horiba Jobin Yvon HR550) equipped with a 532 nm excitation laser, a water immersion objective (Olympus LUMFL, 60×, NA = 1.10), a monochromator (600 grooves per mm grating), and a Synapse CCD detector. A customized 3D-printed *operando* Raman one compartment cell was employed. The working electrode consisted of catalyst-coated CuFeO_2_/Pt thin films, with Ag/AgCl (3 M KCl) as the reference electrode and a Pt counter electrode. Electrolytes of 1 M NaOH (HER condition) and 1 M NaOH + 0.1 M KNO_3_ (NO_3_RR condition) were used to replicate the photoelectrochemical environment. Electrochemical control was provided by a Gamry REF600 potentiostat.

### Product quantification

Electrolyte aliquots were collected at the start and at the end of each electrolysis experiment (and at intermediate times when time-resolved yields were required). Samples were filtered if necessary and stored prior to analysis. All glassware and sampling vials were rinsed thoroughly to avoid contamination, procedural blanks were run for each experiment.

Ammonia (NH_3_/NH_4_^+^) was quantified using the indophenol (Berthelot) colorimetric assay adapted for alkaline media. Briefly, aliquots (2 mL) of the electrolyte collected at the end of each electrolysis experiment were mixed with 2 mL of 1 M NaOH containing 5 wt% salicylic acid and 5 wt% sodium citrate, followed by addition of 1 mL of 0.05 M NaClO (sodium hypochlorite) and 0.2 mL of 1 wt% sodium nitroprusside (C_5_FeN_6_Na_2_O). The mixture was allowed to react for color development. The absorbance was measured at 655 nm using UV-Vis spectroscopy. Calibration curves (see Fig. S1 and S2) were prepared from NH_4_Cl standards in the same electrolyte matrix within a concentration range resembling the experiments conditions. The limits of detection (LOD), the limits of quantification (LOQ), the linear range of the calibration and additional calibration statistics are provided in the SI.

Nitrite (NO_2_^−^) (Fig. S3) and hydroxylamine (NH_2_OH) (Fig. S4) were quantified using the sulfonamide-naphthylamine colorimetric method (absorbance at 540 nm) and the Berg 8-quinolinol method (absorbance at 705 nm), respectively. Calibration procedures, LOD/LOQ and blank corrections are reported in the SI.

Ammonia production rate and faradaic efficiency (FE_NH_3__) were calculated from the molar amount of NH_3_ produced, *n*_NH_3__ (mol), which is obtained from the measured concentration and the sample volume. The ammonia production rate (per geometric area) is given as3
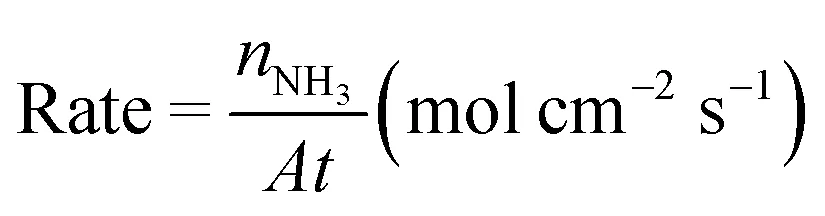
where *A* is the electrode geometric area (cm^2^) and *t* is the electrolysis time (s). The faradaic efficiency for NH_3_ is calculated as4
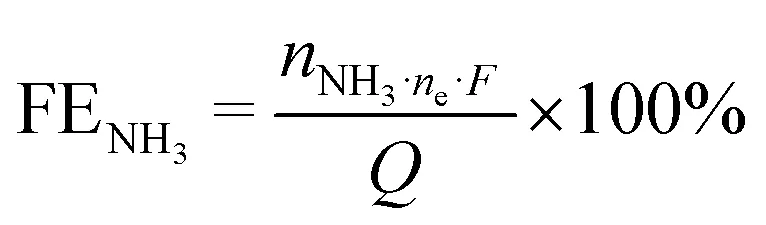
where *n*_e_ is the number of electrons transferred per NH_3_ formed (assumed 8 e^−^ for NO_3_^−^ → NH_3_), *F* is the Faraday constant (96 485 C mol^−1^) and *Q* is the total charge passed during electrolysis (C). All FE values include propagated uncertainties from concentration measurements and charge integration.

### ICP-MS analysis of dissolved metal species

Solution-phase elemental analysis was performed using a Thermo Scientific iCAP RQ ICP-MS equipped with a quadrupole mass analyser and operated in kinetic energy discrimination (KED) mode. Data acquisition and calibration were carried out using QTegra Intelligent Scientific Data Solution software. Calibration curves for Cu, Fe, and Pt were established over the 0–100 ppb range in a 10% HNO_3_ matrix, showing good linearity (*R*^2^ > 0.999) and reproducibility. Electrolyte samples were collected from the catholyte compartment of the custom H-cell after chronoamperometry at 0.63 V *vs.* RHE for 1 h (total volume 40 mL) each aliquot (3–5 mL) was diluted 10 × in 10% HNO_3_ prior to measurement. The concentrations of dissolved Cu, Fe and Pt were determined from the calibration curves and expressed in mg L^−1^ to evaluate time dependent leaching behaviour under photoelectrochemical nitrate reduction conditions.

## Results and discussion

### Cocatalyst screening

CuFeO_2_ films were synthesized *via* an one-step hydrothermal route.^70^ The optimized CuFeO_2_ photocathode thin film was utilized as the starting material to investigate surface modifications with Au, Pt or Co using a post-synthetic hydrothermal procedure performed in 1 M NaOH using a fixed metal ion concentration of 334 µg cm^−2^ (illustrated in Scheme 1, steps 3–4) which was selected as a common baseline to compare Au, Pt, and Co modifications under identical conditions.

**Fig. 1:**
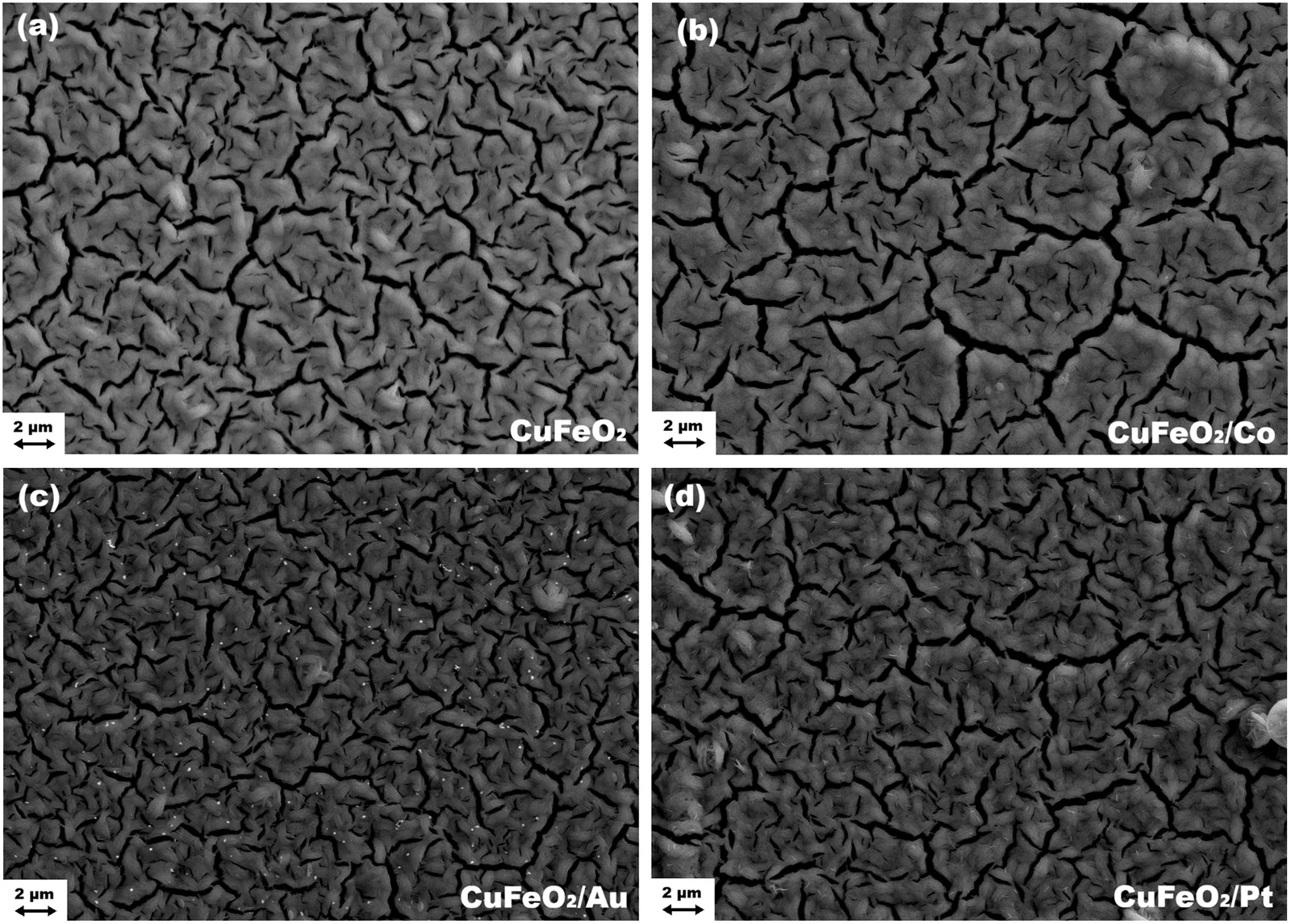
SEM images of the different CuFeO_2_ samples. (a) Bare CuFeO_2_ showing compact agglomerated grains with platelet-like morphology, (b) CuFeO_2_/Co showing no significant morphological changes, (c) CuFeO_2_/Au showing discrete gold nanoparticles (Au NPs), (d) CuFeO_2_/Pt revealing needle-like Pt structures protruding from the surface.

The modified CuFeO_2_ samples were first analysed for their structural and morphological characteristics. Scanning electron microscopy (SEM) analysis revealed distinct morphological differences between unmodified and surface-modified CuFeO_2_ thin films. As previously reported, unmodified CuFeO_2_ are characterized by a uniform microstructure (see Fig. 1a) of compact, agglomerated grains with irregular edges.^70^ These grains exhibited a platelet-like morphology, and the absence of additional features or particle decorations indicated a pristine and well-formed surface.

Modification with cobalt (Co) resulted in negligible changes in the overall morphology of the CuFeO_2_ surface. The grain size and surface texture of CuFeO_2_/Co (Fig. 1b) closely resembled those of the unmodified sample. Energy-dispersive X-ray spectroscopy (EDX) elemental mapping, however, confirmed successful surface enrichment and suggested a homogeneous distribution of Co across the surface (Fig. S5a). The absence of visible and discrete Co-based particles suggests that cobalt modification resulted in the formation a conformal Co-rich surface region rather than individual particles. Gold (Au) modification resulted in more pronounced morphological changes. As evidenced by SEM (Fig. 1c and S5b) Au nanoparticles were formed as indicated by the presence of numerous small, bright spots distributed across the CuFeO_2_ surface. The corresponding EDX mapping verifies the presence of Au nanoparticles (Fig. S5c). The formation of discrete Au nanoparticles, rather than a continuous thin film, likely arises from the pronounced lattice mismatch between Au and CuFeO_2_. Au has an fcc lattice parameter of approximately 4.08 Å,^76^ whereas CuFeO_2_ crystallizes in a rhombohedral (3R) delafossite structure with *a* ≈ 3.03 Å and *c* ≈ 17.1 Å.^77^ This mismatch corresponding to a lateral strain of nearly 35% prevents coherent epitaxial growth, favouring heterogeneous nucleation of isolated Au entities.

The modification of CuFeO_2_ with platinum (Pt) resulted in a distinct morphology that differed significantly from both the unmodified and Au/Co-modified photoelectrodes. Instead of Pt particles, platinum was observed as needle-like or rod-shaped structures (see Fig. 1d) on the surface, which is likely indicative of an anisotropic Pt growth during the hydrothermal deposition process. The UV-Vis absorption measurements of the unmodified and surface-modified CuFeO_2_ photocathodes further supported the successful deposition. For all modified samples (CuFeO_2_/Au, CuFeO_2_/Co, CuFeO_2_/Pt) a higher absorbance across the measured spectral window compared with the bare CuFeO_2_ (Fig. S6) was observed as also frequently reported for other metal modified semiconductors.^78,79^ For the Co-modified sample the largest increase in absorbance supported the formation of a conformal, ultra-thin Co-containing surface layer, which increases optical density and light scattering at the interface. For the Au- and Pt-modified samples, the enhanced absorbance may not only arise from generic metal modification but also from optical resonance effects associated with the discrete nanostructures. For example, nucleated Au and Pt domains can support localized surface plasmon or Mie-type resonances, which enhance local light absorption and near-field intensity. Such resonances have been reported to significantly influence photocatalytic performance in related systems.^80^

**Fig. 2:**
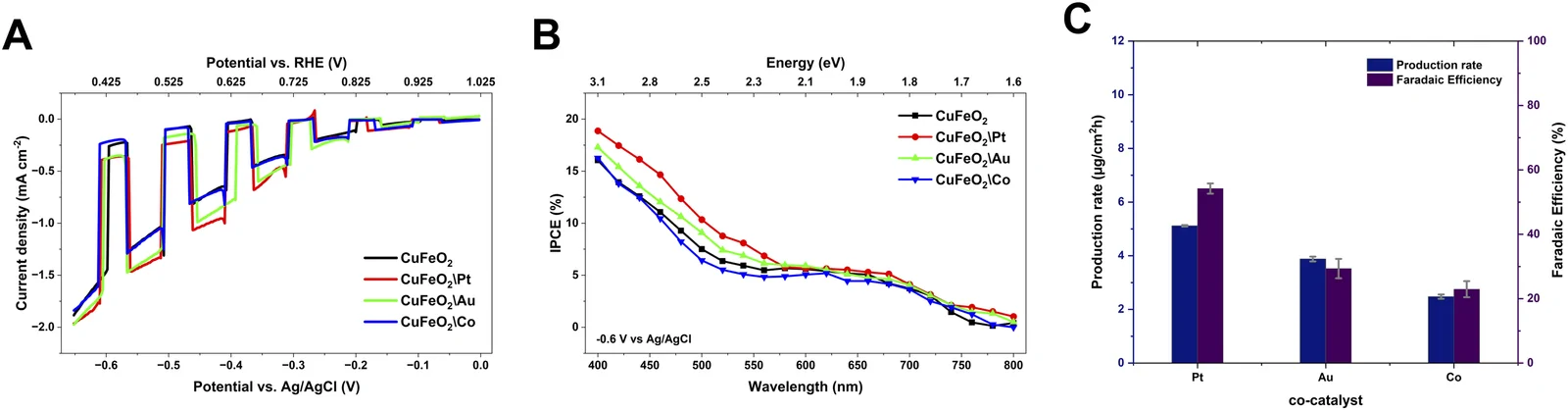
Photoelectrochemical performance of bare and modified CuFeO_2_ photocathodes. (A) Linear sweep voltammetry (LSV) under 1 sun chopped light illumination. (B) Incident photon-to-current efficiency (IPCE) of bare and modified CuFeO_2_ photocathodes measured at a fixed potential of ≈0.4 V *vs.* RHE. (C) faradaic efficiency for NH_3_ and NH_3_ production rate measured at 0.63 V *vs.* RHE (error bars from replicate measurements). All measurements were performed in 100 mM KNO_3_/1.0 M NaOH at 0.63 V *vs.* RHE.

PEC performance was determined in nitrogen-purged electrolytes consisting of 1 M NaOH and 0.1 M KNO_3_. LSV (Fig. 2A) revealed small but still notable differences in photocurrent density between the bare and modified CuFeO_2_ photocathodes. For all samples a photocurrent onset of approximately 0.93 V *vs.* RHE was observed. At 0.4 V *vs.* RHE, the bare CuFeO_2_ electrode exhibited a photocurrent density of −1.7 mA cm^−2^, while with Co modification only a minor improvement (−1.72 mA cm^−2^) was obtained. Modification of the surface with the more noble metals Au and Pt, however, yielded in the generation of higher photocurrent densities of approximately −1.8 mA cm^−2^ at 0.4 V *vs.* RHE. Moreover, for the two noble metal modified CuFeO_2_ photoelectrodes a generally higher photocurrent density was obtained within the potential range 0.7 V–0.4 V *vs.* RHE. Interestingly, comparison of the photoelectrodes in the absence of nitrate suggested that only for the unmodified CuFeO_2_ photoelectrode the low photocurrent onset is maintained (Fig. S7), while a significant shift of at least 100 mV is revealed for the modified electrodes. For the CuFeO_2_/Co and CuFeO_2_/Au also the photocurrent remained lower than for unmodified CuFeO_2_ throughout the potential range measured and only for CuFeO_2_/Pt photoelectrodes at potentials <0.5 V *vs.* RHE higher photocurrents were obtained. While these observations appear to be counterintuitive, in the absence of metal modification photo-induced self-reduction of the unmodified CuFeO_2_ might explain the observations. Nevertheless, the LSV data suggest that Pt modification results in the highest photocurrent generation in the presence of nitrate.

The trends observed in the LSV measurements in the presence of nitrate were consistent with the incident photon-to-current efficiency (IPCE) results obtained at a fixed potential of ≈0.4 V *vs.* RHE (Fig. 2B) using nitrate containing electrolytes. The bare CuFeO_2_ and Co-modified electrodes exhibited comparable IPCE values of approximately 16% at 400 nm illumination, underscoring the limited catalytic impact of Co modification. In contrast, for CuFeO_2_/Au, CuFeO_2_/Pt photocathodes notable improvements within the spectral range of 400–550 nm were obtained with IPCE values reaching 17.3% for CuFeO_2_/Au and 18.9% for CuFeO_2_/Pt at 400 nm illumination. Complementary IPCE spectra recorded across different wavelengths and applied potentials are provided in Fig. S8, further illustrating the wavelength-dependent enhancement in photon-to-electron conversion efficiency particularly for Au- and Pt-modified photoelectrodes.

The selectivity of the photocathodes for nitrate conversion was finally assessed by chronoamperometry and quantification of liquid products. Measurements were conducted under 1 sun illumination at 0.63 V *vs.* RHE (representative CA traces in Fig. S9) and revealed significant differences in faradaic efficiency Fig. 2C and stability. For unmodified CuFeO_2_ no liquid p-NO_3_RR products were detected. Among the modified photoelectrodes, however, irrespectively of the metallic cocatalyst, ammonia formation was observed. For CuFeO_2_/Pt an FE of 54% towards ammonia was revealed, whereas for CuFeO_2_/Au and CuFeO_2_/Co an FE_NH_3__ of ≈29% and ≈21% were determined. Importantly, neither NO_2_^−^ nor NH_2_OH formation was not observed for any of the photoelectrodes tested. Therefore, nitrate conversion using cocatalyst-modified CuFeO_2_-based photoelectrodes generally exhibited good selectivity for ammonia formation under the conditions studied here. Comparison with other reports on p-NO_3_RR (Tables S2 and S3) even underscore the superior selectivity of CuFeO_2_-based electrodes. Importantly, based on the current–time profiles the stability of the modified photoelectrodes appeared to be enhanced after metal modification when compared to the unmodified CuFeO_2_.

**Fig. 3:**
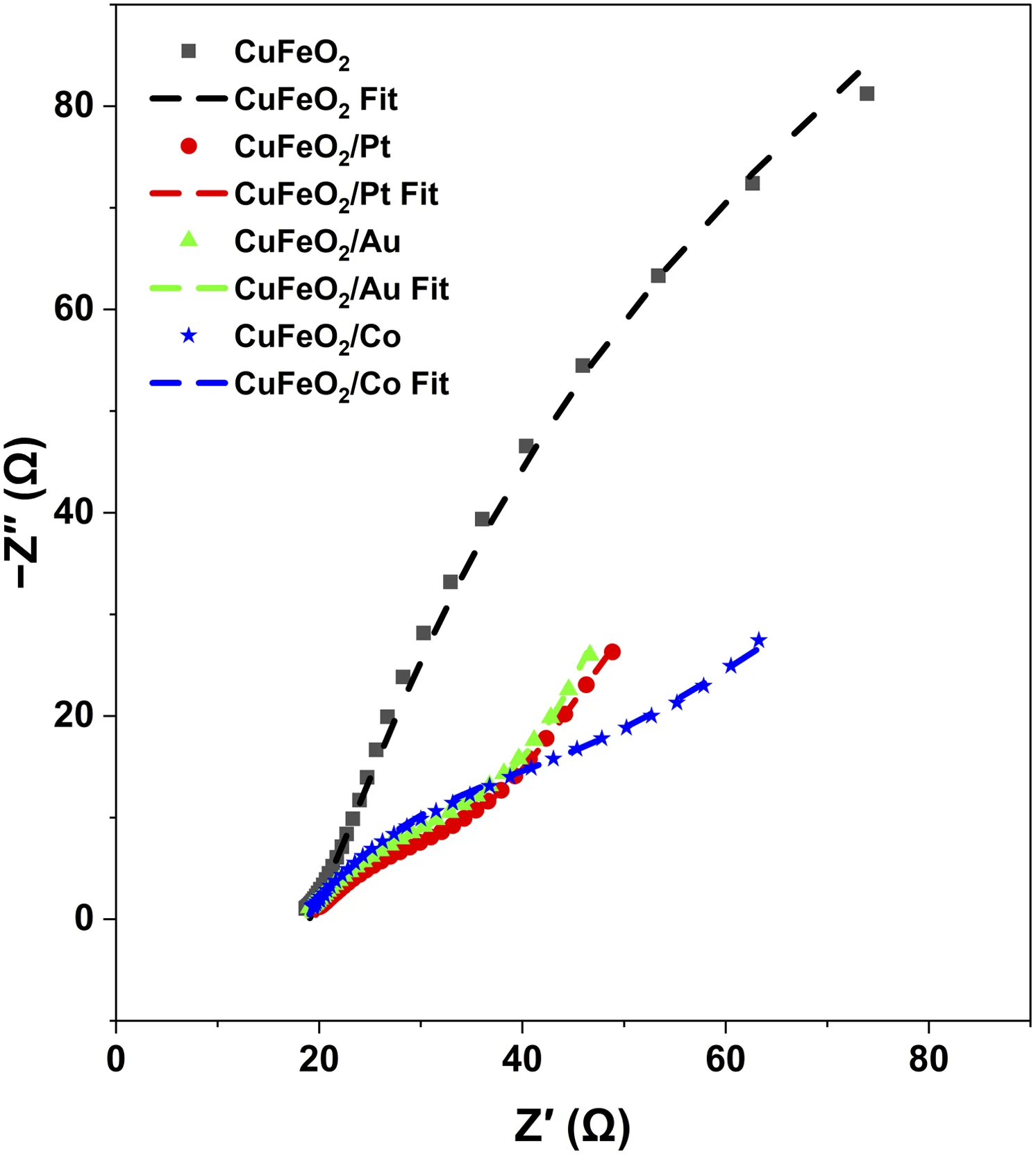
Nyquist plots of pristine and modified CuFeO_2_ photocathodes recorded at 0.63 V *vs.* RHE in 100 mM KNO_3_/1.0 M NaOH under 1 sun illumination. Symbols represent the experimental EIS data, while dashed lines show the fits obtained using a two-time-constant equivalent-circuit model. The corresponding fitting parameters are summarized in Table S1.

To gain insight into how cocatalyst modification affects the interfacial impedance response of CuFeO_2_ based photocathodes under p-NO_3_RR conditions, EIS measurements were performed at 0.63 V *vs.* RHE in 100 mM KNO_3_, 1.0 M NaOH under 1 sun illumination. The Nyquist plots (Fig. 3) show the experimental and fitted EIS data of pristine and cocatalyst modified CuFeO_2_ photocathodes. Bode magnitude plots are provided in the SI (Fig. S10) as a complementary frequency-domain representation of the same impedance data. DRT analysis (Fig. S11) was used to deconvolute the impedance spectra into relaxation time contributions and to guide the choice of the equivalent-circuit model. All samples were fitted using a two-time-constant equivalent circuit model comprising *R*_0_ assigned to the solution resistance, while two *R*_*x*_‖CPE_*x*_ (*x* = 1, 2) elements were assigned to distinguishable faster and slower relaxation contributions within the measured frequency range. The fitted parameters are summarized in Table S1. The Nyquist plot of pristine CuFeO_2_ revealed a substantially larger impedance response dominated mainly by *R*_2_ (1 MΩ). Nevertheless also *R*_1_ was found to be the largest for the pristine CuFeO_2_ photoelectrode compared with the cocatalyst modified versions. *R*_0_ remained nearly constant across all samples, indicating comparable uncompensated solution resistance at the applied potential. Among the cocatalyst modified photocathodes, CuFeO_2_/Pt exhibits the lowest *R*_2_ (131.1 Ω), followed by CuFeO_2_/Au (217.7 Ω) and CuFeO_2_/Co (650.6 Ω). In contrast, *R*_1_ is lowest for CuFeO_2_/Co (16.7 Ω), followed by CuFeO_2_/Pt (27.6 Ω) and CuFeO_2_/Au (34.6 Ω). However, *R*_1_ is considerably smaller than *R*_2_ for all modified electrodes and therefore makes a comparatively minor contribution to the sum of fitted resistive contributions. Considering both contributions, *R*_1_ + *R*_2_ follows the sequence CuFeO_2_/Pt (158.7 Ω) < CuFeO_2_/Au (252.3 Ω) < CuFeO_2_/Co (667.3 Ω). This trend correlates with the determined faradaic efficiency for NH_3_ formation, *i.e.* for CuFeO_2_/Pt the highest FE_NH_3__ was obtained followed by Au and Co containing photoelectrodes (Fig. 2C). EIS is therefore used as complementary evidence for differences in interfacial dynamics under NO_3_RR conditions, whereas FE_NH_3__ remains the direct measure of ammonia selectivity.

### Post-PEC characterization

Building on the activity, selectivity and characterization data presented above, the operational stability of the modified CuFeO_2_ photoelectrodes was further assessed. The bare CuFeO_2_ photocathode exhibited rapid deactivation, which was confirmed by LSV performed after p-NO_3_RR. As shown in Fig. S12, pristine CuFeO_2_ was completely deactivated with the post-PEC LSV trace showing only a negligible photocurrent. In contrast, all surface-modified electrodes retained measurable activity, confirming their superior resilience. Although the photocurrent density decreased during operation, the preserved onset potentials and current profiles indicate that Pt, Au, and Co modifications resulted in a notable stabilization of the catalytic interfaces. The photocurrent behaviour was also found to be in good agreement with visual inspection of the photoelectrodes. A distinct colour change from black to brown was revealed only for the unmodified CuFeO_2_ (Fig. S13) highlighting the irreversible degradation of the photoelectrode during p-NO_3_RR. This instability aligns with previous observations of the intrinsic structural and chemical vulnerabilities of CuFeO_2_ under prolonged PEC operation.^81,82^ For all surface-modified photocathodes, the colour of the photoelectrodes remained unchanged.

To further investigate the stability of the modified photoelectrodes, X-ray photoelectron spectroscopy (XPS) and SEM characterization were performed before and after p-NO_3_RR. XPS analysis of pristine and modified CuFeO_2_ photocathodes (Fig. S14) confirmed the presence of all expected elements, including Cu, Fe, O, and the metals used for surface modification (Au, Co, and Pt). XPS data of the pristine CuFeO_2_ used as the baseline in this study have been reported in detail elsewhere.^70^ In brief, the Cu 2p region is dominated by a Cu^+^ 2p_3/2_ contribution, accompanied by a very weak satellite at ∼945 eV being consistent with the monovalent oxidation state of copper (Cu^+^). The Fe 2p region consisted of a doublet at ∼710.9 eV (Fe 2p_3/2_) and ∼724.4 eV (Fe 2p_1/2_) caused by spin-orbital splitting with multiplet splitting characteristic of high-spin Fe^3+^ in an octahedral coordination environment. The absence of a shoulder at lower binding energies confirmed the absence of Fe^2+^ species. The O 1s spectrum exhibited a broad peak centered at ∼530.1 eV, consistent with lattice oxygen within a complex oxide structure.

**Fig. 4:**
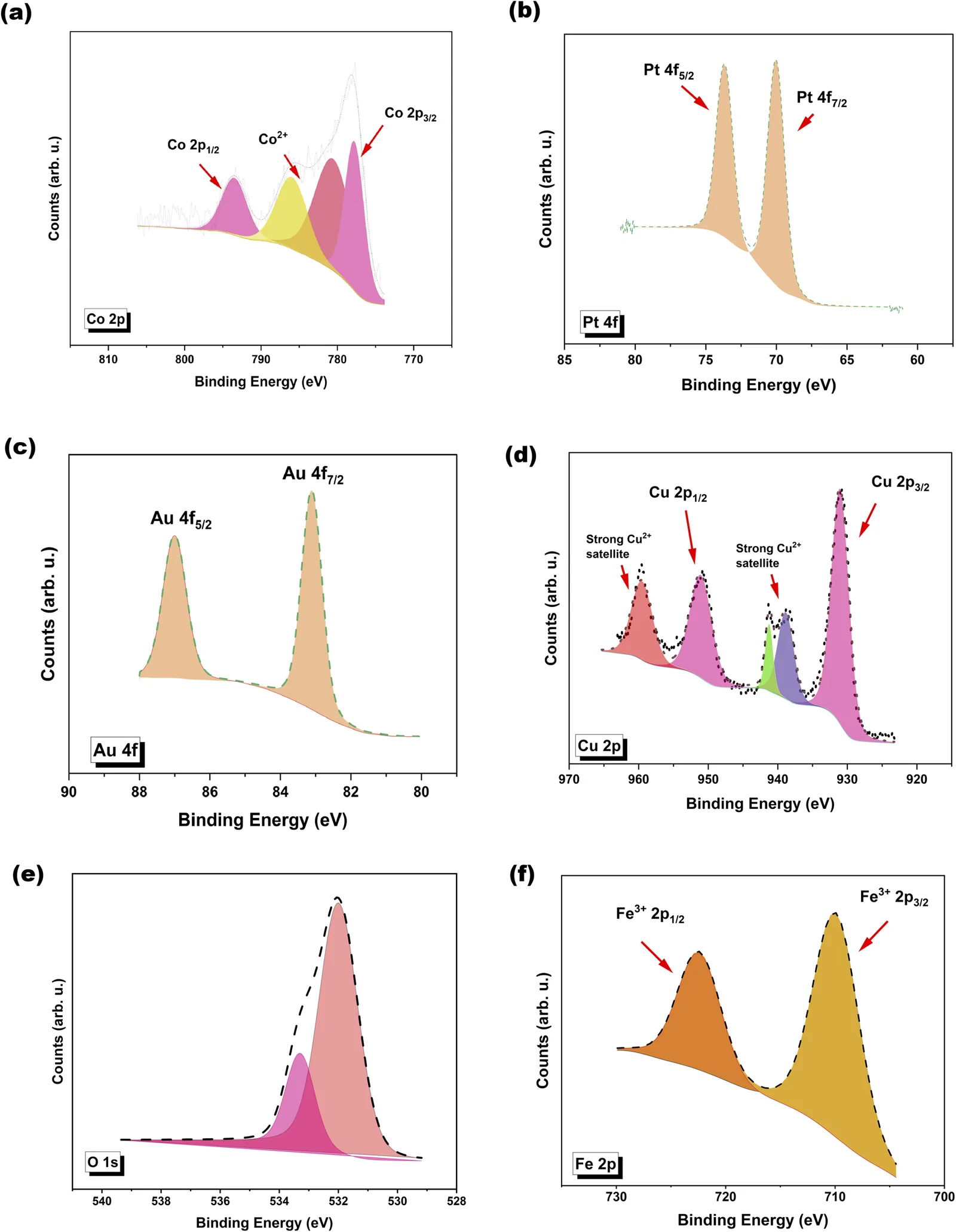
High-resolution XPS spectra of modified CuFeO_2_ surfaces recorded before p-NO_3_RR. (a) Co 2p region for CuFeO_2_/Co, (b) Pt 4f region for CuFeO_2_/Pt, (c) Au 4f region for CuFeO_2_/Au, (d) Cu 2p, (e) O 1s, and (f) Fe 2p regions obtained for CuFeO_2_/Pt. The Cu 2p, O 1s, and Fe 2p spectra shown here are representative for all modified samples and negligible variations among the different metal modified CuFeO_2_ photoelectrodes were observed.

Contrasting the XPS data obtained for pristine CuFeO_2_, upon surface modification distinct chemical changes were observed. For CuFeO_2_/Co, the Co 2p_3/2_ spectrum (Fig. 4a) featured broad peaks with satellite structures, indicative of high-spin Co^2+^. For CuFeO_2_/Pt, the high-resolution Pt 4f spectrum (Fig. 4b) is dominated by metallic Pt^0^ as indicated by Pt 4f_7/2_ and Pt 4f_5/2_ peaks at 70.30 eV and 74.57 eV, respectively.^83^ Similarly for CuFeO_2_/Au, the Au 4f spectrum (Fig. 4c) revealed the presence of metallic Au (peaks at 83.9 eV (4f_7/2_) and 87.6 eV (4f_5/2_)).^84^ The reduced surface state of Pt nanoparticles is presumably advantageous for interfacial charge transfer and likely the catalytic activity.^53^

Interestingly, significant changes were observed in the Cu 2p (Fig. 4d) and O 1s (Fig. 4e) region spectra of all modified electrodes when compared with the pristine CuFeO_2_ photoelectrode. The Cu 2p, O 1s, and Fe 2p region spectra recorded for CuFeO_2_/Pt are shown in Fig. 4d–f (as a representative example of all metal modified CuFeO_2_ photoelectrodes as nearly identical for spectra were obtained for CuFeO_2_/Co and CuFeO_2_/Au). The Cu 2p region of the modified samples displayed a strong satellite contribution, indicative of Cu^2+^ species, in addition to the Cu^+^ signal observed for unmodified CuFeO_2_. This transformation from Cu^+^ to Cu^2+^ is likely related to a surface oxidation caused by the treatment in strong alkaline conditions (1 M NaOH, pH ∼ 13.2) during the performed hydrothermal surface modification. Alongside the changes in the Cu 2p spectra, also a shoulder at high binding energy (∼531.5 eV) is observed in the O 1s spectra that can be attributed to surface hydroxides formed during hydrothermal modification. Despite the significant changes in the O 1s and Cu 2p region spectra, the Fe 2p contribution (Fig. 4f) closely resembles the spectra obtained for unmodified CuFeO_2_ photoelectrodes suggesting that the Fe^3+^ is predominantly present at the surface, which thus remained stable under the alkaline hydrothermal conditions used for modification.

After 1 hour operation under p-NO_3_RR conditions (Fig. S15a–f) the oxidation state of almost all elements remained unchanged and only for Fe notable changes were observed. The Fe 2p spectra of all modified samples Fig. S15f reveal clear changes in oxidation state. Prior to PEC operation, peak fitting indicated exclusively Fe^3+^ contributions (Fe^3+^ = 100 at%). After PEC operation, deconvolution showed the emergence of Fe^2+^ species (Fe^2+^ = 44 at%), while the Fe^3+^ fraction decreased to = 56 at%. This corresponds to an Fe^2+^/Fe^3+^ ratio of 0.79 after PEC, confirming reduction of Fe^3+^ → Fe^2+^. In contrast, the Cu 2p (Fig. S15d) and O 1s (Fig. S15e) spectra of CuFeO_2_/Pt remained unchanged. Importantly, the Pt 4f (Fig. S15b), Co 2p (Fig. S15a), and Au 4f (Fig. S15c) spectra retained their initial metallic or oxide signatures, indicating minimal cocatalyst restructuring. SEM imaging (Fig. S16a–c) corroborated these findings, showing no significant morphological degradation after operation.

Given the significant changes of observed for the unmodified CuFeO_2_, which clearly reduced to metallic Cu, it appears that the enhanced stability of the modified photocathodes might be caused by the formation of surface Cu^2+^ during the hydrothermal deposition of cocatalysts. The reduction of Cu^+^ to Cu^0^ is thermodynamically more favourable, with a reduction potential of 0.521 V *vs.* SHE, with the reduction potential of Cu^2+^ to Cu^0^ being significantly lower (0.34 V *vs.* SHE).^85^ The formation of surface Cu^2+^ in the modified electrodes thus presumably resulted in the formation of a protective layer that resists reduction, considering that the reduction potential of Cu^2+^ is outside the operational range of p-NO_3_RR. While, it can thus be proposed that the observed change in surface oxidation state suppresses the degradation pathway frequently observed for bare CuFeO_2_, where rapid reduction of Cu^+^ leads to structural and chemical instability, a generally favourable electron transfer to the metallic cocatalysts might explain the enhanced stability. In addition, the surface modification fundamentally alters the coordination environment of the copper active sites. In the unmodified delafossite structure, the surface is dominated by linearly coordinated Cu^+^ ions (O–Cu^+^–O), a fragile configuration that collapses readily under cathodic potentials, leading to structural degradation and reduction into metallic Cu^0^. By contrast, the modified surface stabilizes Cu^2+^, which results in four to six-fold oxygen coordination. This higher coordination increases the energy required to break Cu–O bonds and shifts localized reduction potentials far beyond the operational p-NO_3_RR window. Moreover, the high local OH^−^ concentration passivates the oxide surface and results in a higher stability of the photoelectrode.^86^

For the CuFeO_2_/Pt photoelectrode also a structural integrity was revealed by cross-sectional SEM imaging performed before and after performance evaluation. The layered structure, film thickness, and interface quality remained unchanged after operation, indicating excellent morphological stability (Fig. S17). Raman spectroscopy was additionally employed to verify the structural integrity of the CuFeO_2_/Pt photocathodes before and after photoelectrochemical operation (Fig. S18). For CuFeO_2_/Pt the characteristic *E*_g_ (∼342 cm^−1^) and *A*_1g_ (∼710 cm^−1^) vibrational modes of the 3R-CuFeO_2_ phase are revealed, corresponding to Cu–O stretching and O–Cu–O bending, respectively.^87^ The persistence of these bands after operation confirms that the delafossite structure remains largely intact. Nevertheless, a new feature appearing near 294 cm^−1^ in the post-catalysis spectrum can be assigned to a Fe_2_O_3_-related vibration mode,^88^ consistent with partial reduction of Fe^3+^ to Fe^2+^ as also observed by XPS. Thus, addressing the partial reduction of iron appears to be pivotal to further improve Cu-delafossite photoelectrode stability. The incorporation of an additional protective overlayer or the development of an alternative stabilization strategy will be critical for further improving the long-term performance of CuFeO_2_-based photocathodes.

To complement the surface stability assessment, solution-phase elemental analysis was performed by ICP-MS after chronoamperometry at 0.63 V *vs.* RHE using CuFeO_2_/Pt photocathode. Table 1 summarizes the concentrations of dissolved Cu, Fe and Pt detected in the catholyte. The Cu and Fe contents increased modestly with operation time, reaching 0.64 mg L^−1^ and 0.11 mg L^−1^ after 1 h, respectively, whereas Pt showed only a slight increase from 0.023 to 0.031 mg L^−1^. For the 40 mL electrolyte volume, this corresponds to dissolution of 9 µg Cu, 3 µg Fe, and 0.3 µg Pt. Relative to the CuFeO_2_ film mass and the Pt loading (500 µg cm^−2^), these values represent <0.07% and <0.04% losses, respectively, confirming that leaching is negligible and that Pt remains predominantly at the electrode surface.

**Table 1:** Quantitative ICP-MS analysis of CuFeO_2_/Pt samples showing elemental concentrations of Pt, Fe, and Cu after dilution (0.1×) in 10% HNO_3_. Values are reported as measured intensities and corresponding concentrations (mg L^−1^) in the analysed suspensions

Sample	Cu (mg L^−1^)	Fe (mg L^−1^)	Pt (mg L^−1^)
CuFeO_2_/Pt (blank)	0.417	0.048	0.023
CuFeO_2_/Pt (1 h)	0.641	0.112	0.031

### Process conditions and material optimization

Subsequent optimization efforts of the p-NO_3_RR performance were focused on utilizing on CuFeO_2_/Pt, which was shown to provide efficient charge extraction, high ammonia selectivity, and extended structural stability, rendering it the most promising platform for advancing PEC nitrate to ammonia conversion with CuFeO_2_ photoelectrodes.

Potential-dependent measurements (Fig. S19 and S20) revealed the highest FE_NH_3__ at 0.63 V *vs.* RHE. Although the absolute NH_3_ production rate increases at more negative potentials, the FE declines (*e.g.*, at 0.53 V *vs.* RHE) due to enhanced competing hydrogen evolution and presumably enhanced degradation. Moreover, concentration-dependent measurements using CuFeO_2_/Pt indicated that both the FE_NH_3__ and the production rate saturate at an initial concentration of ≈100 mM NO_3_^−^, consistent with substrate-coverage or mass-transport limitations beyond this concentration range which is likely the result of the measurement setup/geometry. Thus, optimization of Pt loading and the Pt deposition procedure were determined using chronoamperometry performed at 0.63 V *vs.* RHE using electrolytes containing 100 mM NO_3_^−^.

**Fig. 5:**
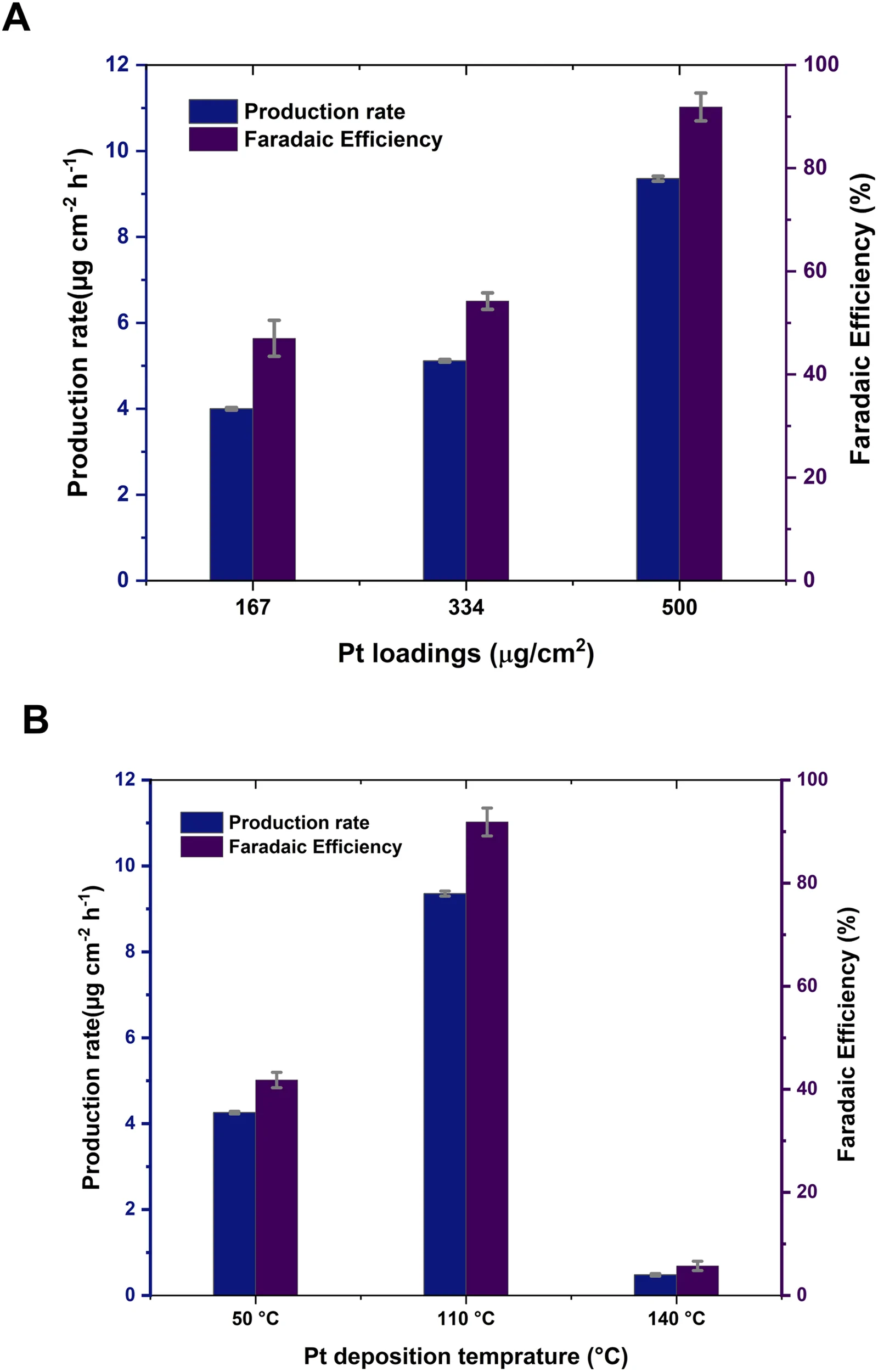
Comparative ammonia production and faradaic efficiency for CuFeO_2_/Pt photocathodes. (A) Effect of Pt cocatalyst loading (167, 337, 500 µg cm^−2^), (B) Influence of Pt deposition temperature on NH_3_ faradaic efficiency and production rate (representative temperatures: 50, 110, 140 °C). The NH_3_ faradaic efficiency and production rate are obtained at 0.63 V *vs.* RHE.

Three Pt loading were tested (167, 337 and 500 µg cm^−2^) and the deposition temperature was varied between 50 and 140 °C (Fig. 5). With increasing Pt loading higher photocurrents were observed as a result of an improved charge-carrier separation. Nevertheless, a non-monotonic trend was observed for the FE_NH_3__ and at the highest tested loading and optimized deposition conditions (110 °C) the maximum FE_NH_3__ of ≈92% was reached.

To confirm that the detected ammonia originates from electrochemical nitrate reduction rather than adventitious contamination, we performed control experiments in nitrate-free electrolyte (1 M NaOH) Fig. S21. Under these conditions, a photocurrent was sustained, which can be attributed to the hydrogen evolution reaction (HER), but no indophenol absorbance peak at 655 nm was observed. In contrast, the optimized CuFeO_2_ photocathode in nitrate containing electrolyte exhibited both a clear 655 nm absorbance peak and higher photocurrent. The detection limit of the indophenol assay was determined to be 1.95 µM, whereas the optimized sample produced 27.8 µM NH_4_^+^ within 1 h (10 µg cm^−2^ h^−1^ in 20 mL electrolyte), 14× above the detection limit and well beyond typical contamination levels reported in the literature (1–10 µM).^73^ These results demonstrate that the observed ammonia production is driven by photoelectrochemical nitrate reduction and not by background contamination.

### Mechanistic considerations

CuFeO_2_/Pt were shown to enable selectivity for ammonia formation from nitrate reduction at potentials of 0.63 V *vs.* RHE. Comparison to recent literature (Tables S2 and S3) reveals the superior performance of the photoelectrodes prepared within this study, as other reported photoelectrodes predominately favour nitrite over ammonia production. *Operando* Raman measurements under varying electrochemical conditions (532 nm excitation) provide evidence of a tandem hydrogenation mechanism and the co-catalytic participation of the modified oxide matrix (Fig. S22). At open-circuit potential (OCP), a band at 1647 cm^−1^ representative of the *δ*(H–O–H) bending vibration of interfacial water molecules is observed.^89^ Under HER bias in 1 M NaOH, the same water band at 1647 cm^−1^ is observed together with a shoulder peak at 2100 cm^−1^ that might indicate the existence of Pt–H (stretching vibration *ν*_Pt–H_),^89^ suggesting the presence of chemisorbed atomic hydrogen (*H*_ads_) on Pt. Crucially, upon addition of nitrate (p-NO_3_RR condition), new interfacial species appeared: a band at 1364 cm^−1^ corresponding to the *ν*_3_ asymmetric stretching of coordinated nitrate/nitrite, and a *ν*_1_ symmetric stretch around 1050 cm^−1^.^90,91^ The simultaneous presence of the steady-state Pt–H peak at 2100 cm^−1^ alongside nitrogenous intermediates at 1050 and 1364 cm^−1^ likely indicate that p-NO_3_RR occurs *via* a H_(ads)_-mediated mechanism (pathway A in Fig. S23). Thus, it is proposed that Pt does not act as a standalone catalyst, instead, it supplies atomic hydrogen that migrates *via* spillover to reduce nitrogen species dynamically chemisorbed on adjacent Cu–Fe active sites resulting in a maximum FE for ammonia of ≈92% at 0.63 V *vs.* RHE under 1 sun illumination with the optimized CuFeO_2_/Pt photocathode. Nevertheless, further mechanistic studies are required to full disclose the reaction mechanism.

## Conclusions

This study demonstrates the potential of CuFeO_2_-based photocathodes for photoelectrochemical (PEC) nitrate reduction (NO_3_RR) under solar illumination. Surface modifications with platinum (Pt), gold (Au), and cobalt (Co) were systematically explored and among the modified systems, CuFeO_2_/Pt facilitated the formation of ammonia at a faradaic efficiency (FE) of 92% even at 0.63 V *vs.* RHE. Complementary EIS analysis independently showed that CuFeO_2_/Pt exhibits the least resistive interfacial response among the modified photocathodes, with the total fitted resistance (*R*_1_ + *R*_2_), dominated by *R*_2_, following the sequence Pt < Au < Co, consistent with the FE_NH_3__ ordering. Importantly, surface functionalization was shown to result in the formation of surface Cu^2+^ which is assumed to be pivotal in stabilizing the photocathode by suppressing the reduction of Cu^+^ to Cu^0^. Thus, irrespectively of the surface modification operational stability of CuFeO_2_-based photocathodes was enhanced compared to unmodified CuFeO_2_. Despite these advancements, stability challenges remain, particularly concerning the partial reduction of Fe^3+^ to Fe^2+^ during prolonged operation, which continues to limit electrode durability. Still, this work establishes CuFeO_2_/Pt as a highly promising photocathode for selective and stable PEC nitrate-to-ammonia conversion at low overpotentials. Moreover, the results presented in this work provide valuable insights into the interplay between cocatalyst identity, semiconductor properties, and reaction conditions, paving the way for future advancements in solar-driven photoelectrochemical nitrate reduction.

## Author contributions

M. E. I.: conceptualization, investigation, methodology, formal analysis, data curation, visualization, writing – original draft, and review & editing. A. K.: investigation and methodology, formal analysis, review & editing. Y. K.: investigation and methodology, formal analysis, review & editing. M. A. A. M.: investigation and methodology, formal analysis, review & editing. M. A.-L.: supervision, resources, review & editing. L. A. supervision, resources, review & editing. B. T. M.: conceptualization, supervision, resources, and writing – review and editing.

## Conflicts of interest

There are no conflicts to declare.

## Supplementary Material

RA-OLF-D6RA05930A-s001

## Data Availability

Data for this article, including raw Raman, XRD, XPS, SECM, GC, and photoelectrochemical measurements together with analysis scripts, are available at Figshare (https://doi.org/10.6084/m9.figshare.33297216). Supplementary information (SI): Fig. S1. See DOI: https://doi.org/10.1039/d6ra05930a.
