# Supplementary material for: Photoelectrochemical nitrate reduction to ammonia on CuFeO2 photocathodes modified with metallic cocatalysts
Source: RSC Adv. 2026 Sep 21;16(44):55859–72. doi: 10.1039/d6ra05930a (PMC13591077; doi:10.1039/d6ra05930a)
Supplement: Supplementary file 1 [file RA-016-D6RA05930A-s001.pdf]

Supplementary Information (SI) for RSC Advances.
This journal is © The Royal Society of Chemistry 2026

# Supporting Information

## Photoelectrochemical Nitrate Reduction to Ammonia on $CuFeO_2$ Photocathodes Modified with Metallic Cocatalysts

Mohamed El Idrissi, Anastasiia Kotova, Ying Kong, Muhammad Adib Abdillah Mahbub, Mohammed Abd-Lefdil, Lahoucine Atourki and Bastian Timo Mei

## Section 1: Experimental details

### Synthesis of $CuFeO_2$ and Surface Modification with Au, Pt and Co

The $CuFeO_2$ films reported here were prepared by a one-step hydrothermal method followed by high-temperature annealing. Fluorine-doped tin oxide (FTO, ≈7 Ω sq−1) substrates (1.0 × 2.0 cm) were cleaned by sequential sonication in detergent, deionized water, acetone and isopropanol. Fresh aqueous precursor solutions were prepared immediately before each run: $CuSO_4·5H_2O$ and $FeSO_4·7H_2O$ were dissolved to give 0.10 M each and urea was added to reach a molar ratio urea: (Cu+Fe) = 4:1. The starting solution pH was measured and documented (typical initial pH ≈ 3.8–3.9). The cleaned FTO substrates were placed FTO-side down in a Teflon (PTFE) liner to favor heterogeneous nucleation on the conductive face, the precursor solution was transferred into the liner to fully immerse the substrates, and the closed autoclave was heated to 110 °C for 12 h. After natural cooling to room temperature the substrates were removed, rinsed thoroughly with deionized water until the rinses were clear, dried under $N_2$ and annealed in air at 650 °C for 1 h using a 5 °C $min^{-1}$ heating ramp. Mechanistically, film formation proceeds via controlled in situ hydrolysis and redox chemistry. Urea decomposes upon heating to slowly release ammonia and hydroxide, producing a mixed (Cu,Fe) hydroxide intermediate $[(Cu,Fe)(OH)_4]$ on the substrate surface, simultaneously $Fe^{2+}$ undergoes oxidation to $Fe^{3+}$ and reduces a fraction of $Cu^{2+}$ to $Cu^+$, enabling formation of the $Cu^+$–$Fe^{3+}$ delafossite framework without an external reducing agent. Maintaining a mildly acidic starting pH is critical for heterogeneous nucleation on the substrate: strongly alkaline baths favor homogeneous nucleation and the formation of dispersed particle suspensions that suppress continuous film growth. Surface modification with Au, Pt and Co was performed by brief hydrothermal depositions from alkaline metal salt baths prepared from concentrated aqueous stock solutions. Stock solutions of $H_2PtCl_6$, $HAuCl_4$ and $Co(NO_3)_2$ were prepared and their concentrations verified, representative measured concentrations were approximately 14 g $L^{-1}$ for $H_2PtCl_6$, 13 g $L^{-1}$ for $HAuCl_4$ and 14.2 g $L^{-1}$ for $Co(NO_3)_2$. For each deposition a measured volume of the selected stock solution was combined with 16 mL of 1.0 M NaOH to produce the alkaline modifier bath, the mixture was stirred for 10 min at room temperature. $CuFeO_2$ coated substrates were immersed FTO side down in the modifier bath in a PTFE liner, sealed in an autoclave and heated to 110 °C for 1 h, then allowed to cool naturally. After deposition the samples were rinsed thoroughly with deionized water and dried under $N_2$.

### Product Quantification

Ammonia ($NH_3$/$NH_4^+$) in catholyte samples was quantified by the indophenol (Berthelot) reaction adapted for alkaline media.[1,2] In this method ammonium is converted to ammonia under alkaline conditions and reacts with hypochlorite to form monochloramine, which then couples with salicylic-derivative phenols under sodium nitroprusside catalysis to yield an indophenol dye with a characteristic blue color. For each analysis 2.0 mL of sample (diluted as required to fall within the calibration range) was mixed with 2.0 mL of phenol reagent prepared by dissolving 5.0 g NaOH, 7.0 g salicylic acid and 15.0 g trisodium citrate in 50 mL water, followed by addition of 1.0 mL sodium hypochlorite solution (14 wt% in water) and 0.20 mL catalyst solution (1 mg sodium nitroprusside per 100 mL water). After colour development the absorbance at 655 nm was measured by UV-vis spectrophotometry. Calibration was performed using $NH_4Cl$ standards prepared in 1.0 M KOH containing $NO^{3-}$ with concentrations of 0.1, 0.5, 1.0, 2.0, 3.0, 5.0 and 10.0 μg $mL^{-1}$; the resulting calibration curve followed y = 0.104x − 0.014 with $R^2$ = 0.999, showing a linear relationship between absorbance and $NH^{4+}$ concentration (representative spectra and calibration are shown in Figure S2).

$$NH_4^+ + H_2O \rightleftharpoons NH_3 + H_3O^+$$

$$NH_3 + NaClO \rightleftharpoons NH_2Cl + NaOH$$

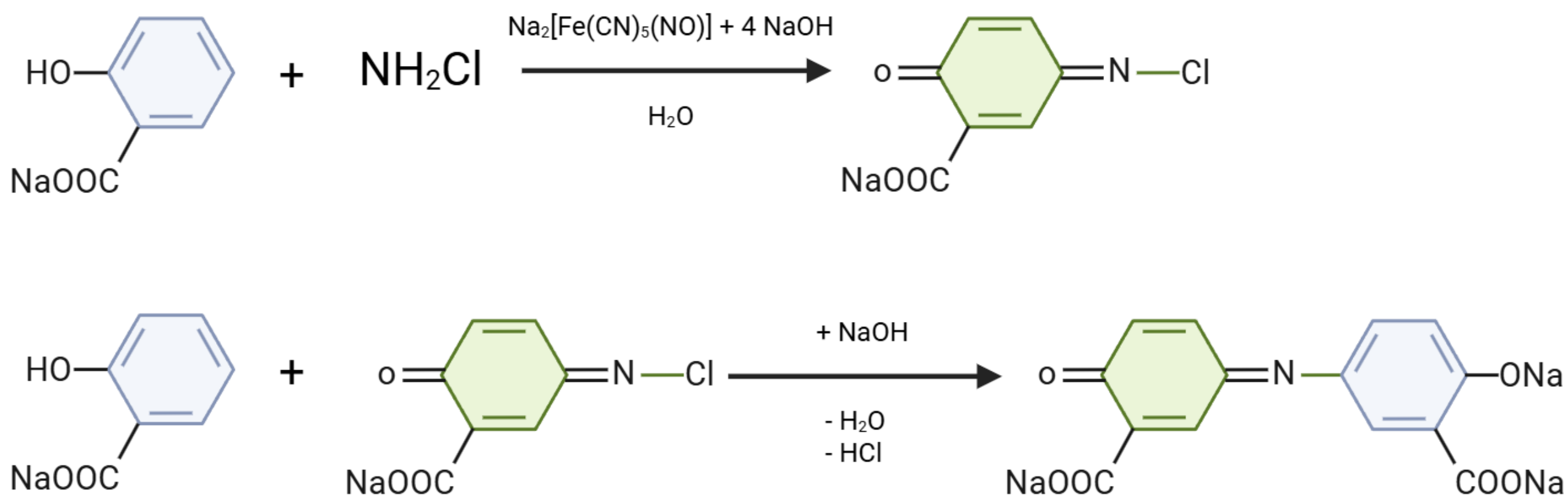


**Figure S1: Reaction scheme for the indophenol (Berthelot) formation: stepwise conversion of ammonium/ammonia to monochloramine and subsequent coupling with salicylic-derivative phenols under sodium nitroprusside catalysis to yield the blue indophenol chromophore measured at 655 nm.**

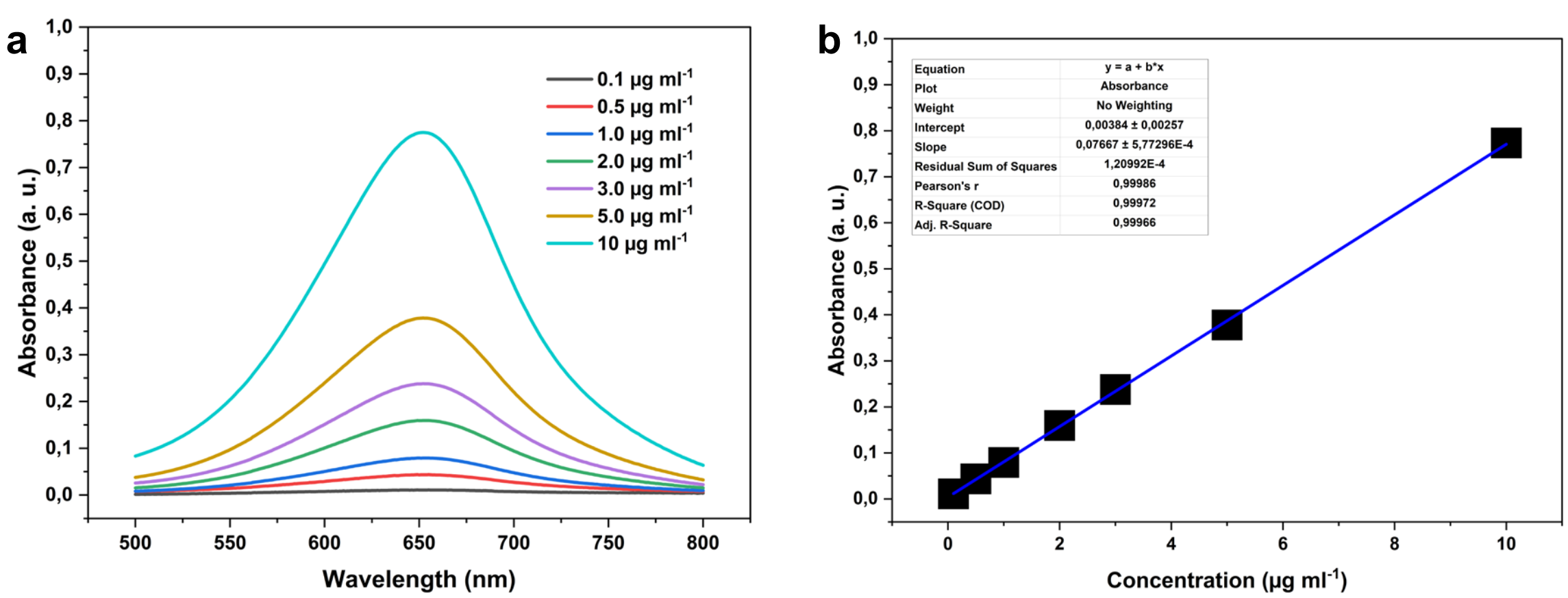


**Figure S2: (a) Representative UV-vis spectra of $NH^+_4$ standards (0.1–10.0 μg $mL^{-1}$) after indophenol color development; (b) calibration curve of absorbance at 655 nm versus $NH_4^+$ concentration with linear fit y = 0.076x + 0.0038 ($R^2$ = 0.999)**

Nitrite ($NO_2^-$) quantification employed the classic sulfonamide/naphthyl-diamine colorimetric reaction.[3,4] A stock sulfonamide reagent was prepared by dissolving 0.5 g sulfonamide in 50 mL of 2.0 M HCl (reagent A), and a color developer solution was prepared by dispersing 20 mg N-(1-naphthyl)ethylenediamine dihydrochloride in 20 mL DI water (reagent B). For each measurement 0.1 mL of reagent A was added to 5.0 mL of standard or appropriately diluted sample and allowed to react for 10 min; 0.1 mL of reagent B was then added, the solution was mixed and allowed to stand for 30 min, and the UV-vis absorbance spectrum from 400 to 640 nm was recorded. The diagnostic absorbance maximum for $NO_2^-$ was taken at 540 nm. Calibration used $KNO_2$ standards at 0.05, 0.1, 0.2, 0.5, 1.0, 2.0 and 3.0 μg $mL^{-1}$ and yielded a linear fit y = 0.768x – 0.012 with $R^2$ = 0.999 (representative spectra and the calibration plot are provided in Figure S10).

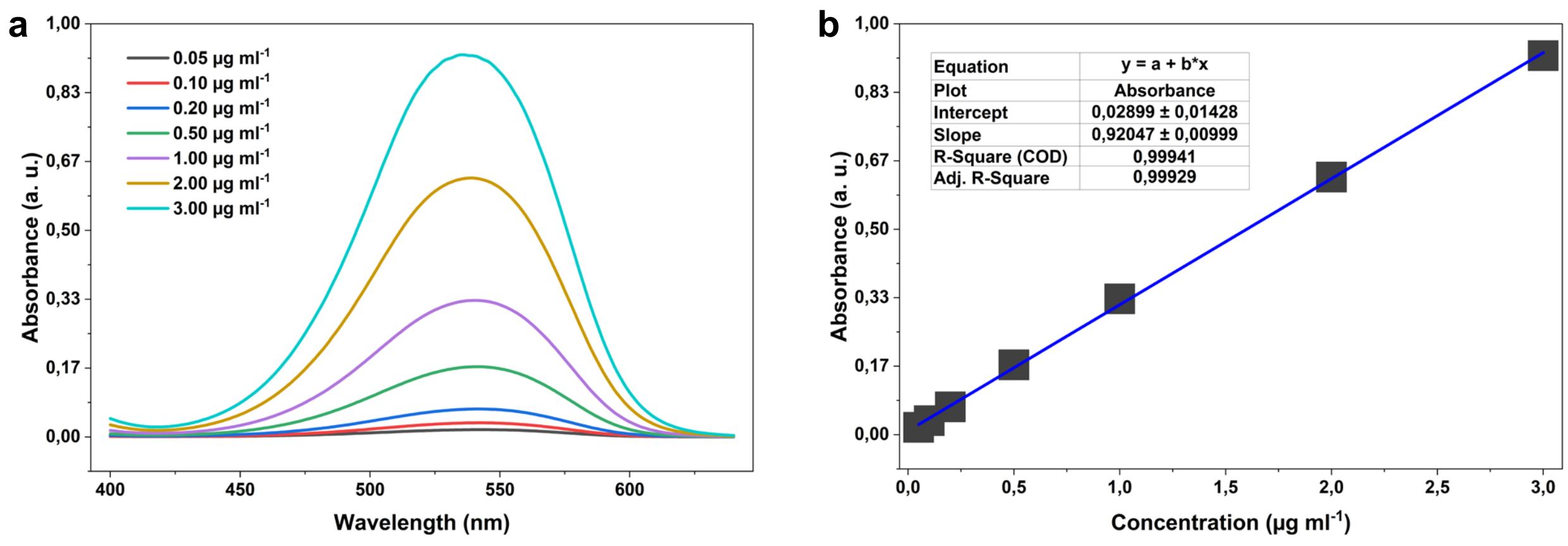


**Figure S3: Representative UV-vis spectra of $NO_2^-$ standards (0.05–3.0 µg $mL^{-1}$) after sulfonamide/naphthyl-diamine color development; (b) calibration curve of absorbance at 540 nm versus $NO_2^-$ concentration with linear fit y = 0.92x – 0.028 ($R^2$ = 0.999).**

Hydroxylamine ($NH_2OH$) was measured photometrically following the Berg protocol based on 8-quinolinol chemistry.[5] In alkaline solution 8-quinolinol reacts with hydroxylamine and, after oxidation and further reaction steps, produces a coloured indo-oxin derivative. For each determination 1.0 mL of analysis solution was mixed with 1.0 mL sodium carbonate solution (106 g sodium carbonate per 1000 mL water) and 1.0 mL 8-quinolinol solution (1 g 8-quinolinol dissolved in 100 mL absolute ethanol). The reaction develops an intense green colour within 10 min; the absorbance was recorded at 705 nm and used for quantification against standards prepared in the same matrix (representative reaction scheme is shown in **Figure S4**).

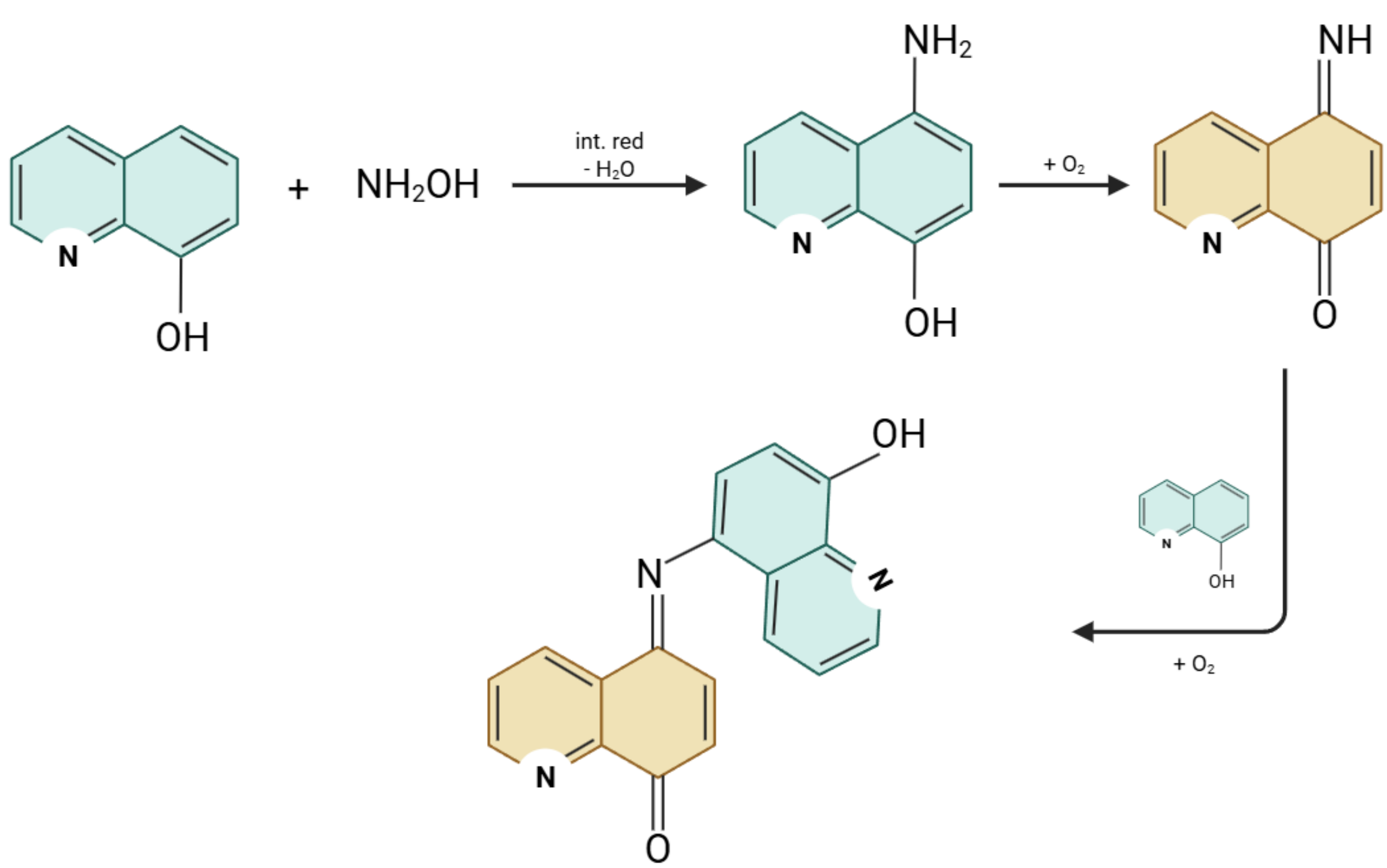


**Figure S4: Reaction scheme for photometric hydroxylamine determination based on 8-quinolinol: nucleophilic addition of hydroxylamine to 8-quinolinol, oxidative conversion to the reactive intermediate and subsequent formation of the colored indo-oxin derivative measured at 705 nm.**

**Stability of $CuFeO_2$**

The operational stability of the optimized $CuFeO_2$ photocathode was evaluated under cathodic photoelectrochemical conditions and summarized here, full datasets, extended analyses and original figures are available in our previous publication (**Ref. 1**). Briefly, the films studied here were grown hydrothermally and annealed under the conditions reported in Ref. 1, Visually and electrically, $CuFeO_2$ films undergo rapid, pronounced changes during cathodic PEC operation. Electrodes change color (dark green → brown) and exhibit a marked increase in electrical conductivity after testing, consistent with the formation of conductive phases at or near the surface. These macroscopic changes correlate with the observed loss of photocurrent and the collapse of effective band bending that supports charge separation in the pristine delafossite. Surface chemical analysis by X-ray photoelectron spectroscopy and Auger measurements reveals the dominant redox transformations. Pristine films show Cu predominantly as $Cu^+$ and Fe as $Fe^{3+}$ with lattice oxygen dominating the O 1s region. After cathodic PEC exposure, Cu is reduced to metallic $Cu^0$ (Cu 2p and Cu LMM signatures), while Fe shows partial reduction toward $Fe^{2+}$ and the O 1s region develops higher-energy components attributable to hydroxides/defect species. These chemical changes indicate that cation reduction and surface hydroxylation are primary chemical markers of degradation. Morphological characterization supports a chemically driven degradation mechanism. Post-PEC SEM images show loss of the original plate-like film morphology, particle agglomeration and surface pitting. The combined chemical and morphological evidence indicates that reduction of $Cu^+ \rightarrow Cu^0$ (complete) and partial $Fe^{3+} \rightarrow Fe^{2+}$ produce conductive domains and structural disorder that short-circuit the semiconductor and introduce recombination centers, explaining the rapid decline in PEC performance. Implications for the present study and data availability. Because these degradation pathways prevent reliable long-term $NO_3$RR testing on unmodified $CuFeO_2$, the current work focuses on modest, well-dispersed surface modifications (Pt, Au, Co) designed to improve interfacial charge transfer, stabilize surface chemistry, and limit the formation of metallic Cu domains. For readers seeking full spectra, peak-fit parameters, raw XPS/Auger data, extended SEM series and additional control experiments, please consult **Ref. 1** and its Supporting Information.

**Pt loading calculation**

The cocatalyst loadings reported (167, 334 and 500 μg·cm$^{-2}$) represent values derived from precursor concentration and electrode area. The platinum loading was calculated using:

$$L_{pt} = \frac{V_{Pt} \times C_{stock} \times f_{Pt} \times \eta}{A}$$

Where $L_{Pt}$ is the Pt loading (mg·cm$^{-2}$), $V_{Pt}$ is the volume of $H_2PtCl_6$ stock solution added (mL), $C_{stock}$=14 mg mL$^{-1}$ is the concentration of the stock solution, $f_{Pt}$ = 0.4765 is the mass fraction of Pt in anhydrous $H_2PtCl_6$, $\eta$ is the deposition efficiency, and $A$ = 2 cm$^2$ is the geometric electrode area.

To estimate $\eta$, deposited $CuFeO_2$/Pt films were mechanically removed from FTO substrates, weighed as powders, and analyzed by XPS survey spectra. The relative Pt atomic fraction obtained from XPS was combined with the measured total film mass to approximate the actual Pt content. Comparison with the theoretical precursor input indicated an effective deposition efficiency of 20%. Using this relation, the calculated Pt loadings were approximately 0.167, 0.334, and 0.500 $\mu$g·cm$^{-2}$ for $V_{Pt}$ = 0.25, 0.50, and 0.75 mL, respectively.

The same volume based calculation approach was applied for Au and Co cocatalysts, using the corresponding precursor concentrations and mass fractions, with deposition efficiency estimated analogously.

**Section 2: Supplementary data**

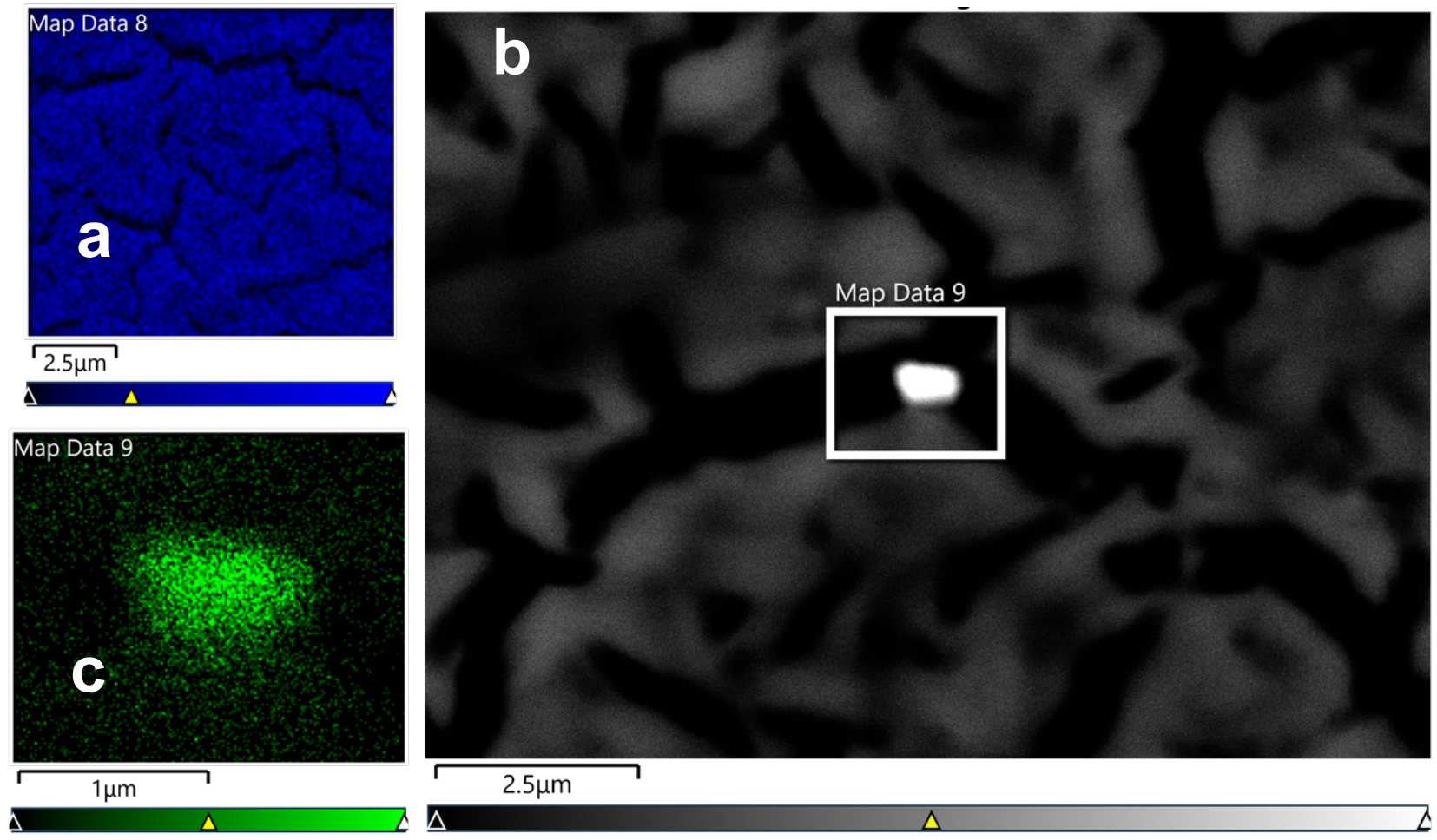


**Figure S5: Complementary SEM and EDX analyses. (a) EDX elemental map of Co-modified $CuFeO_2$ demonstrating uniform Co coverage across the imaged area. (b) High-magnification SEM of Au-modified $CuFeO_2$ highlighting Au nanoparticle distribution. (c) Corresponding EDX map confirming Au presence at the nanoparticle sites.**

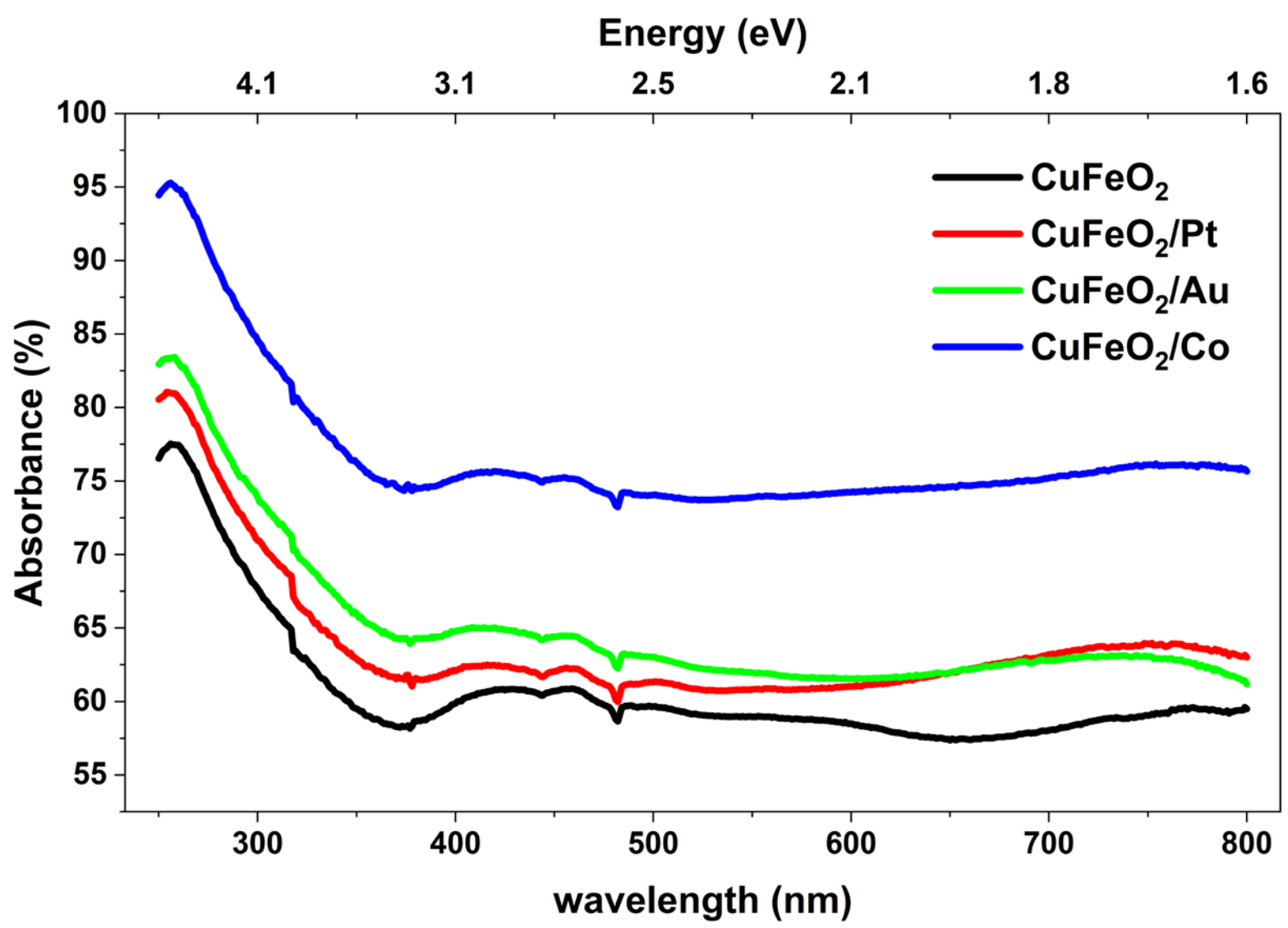


**Figure S6: UV-vis absorption spectra of $CuFeO_2$ and surface modified $CuFeO_2$ photocathodes. For the Co-modified photoelectrode an increase in absorbance across the visible range is observed that is consistent with a thin, conformal Co surface layer observed by SEM/EDX, Au and Pt modifications resulted in a modest enhancement in the absorbance.**

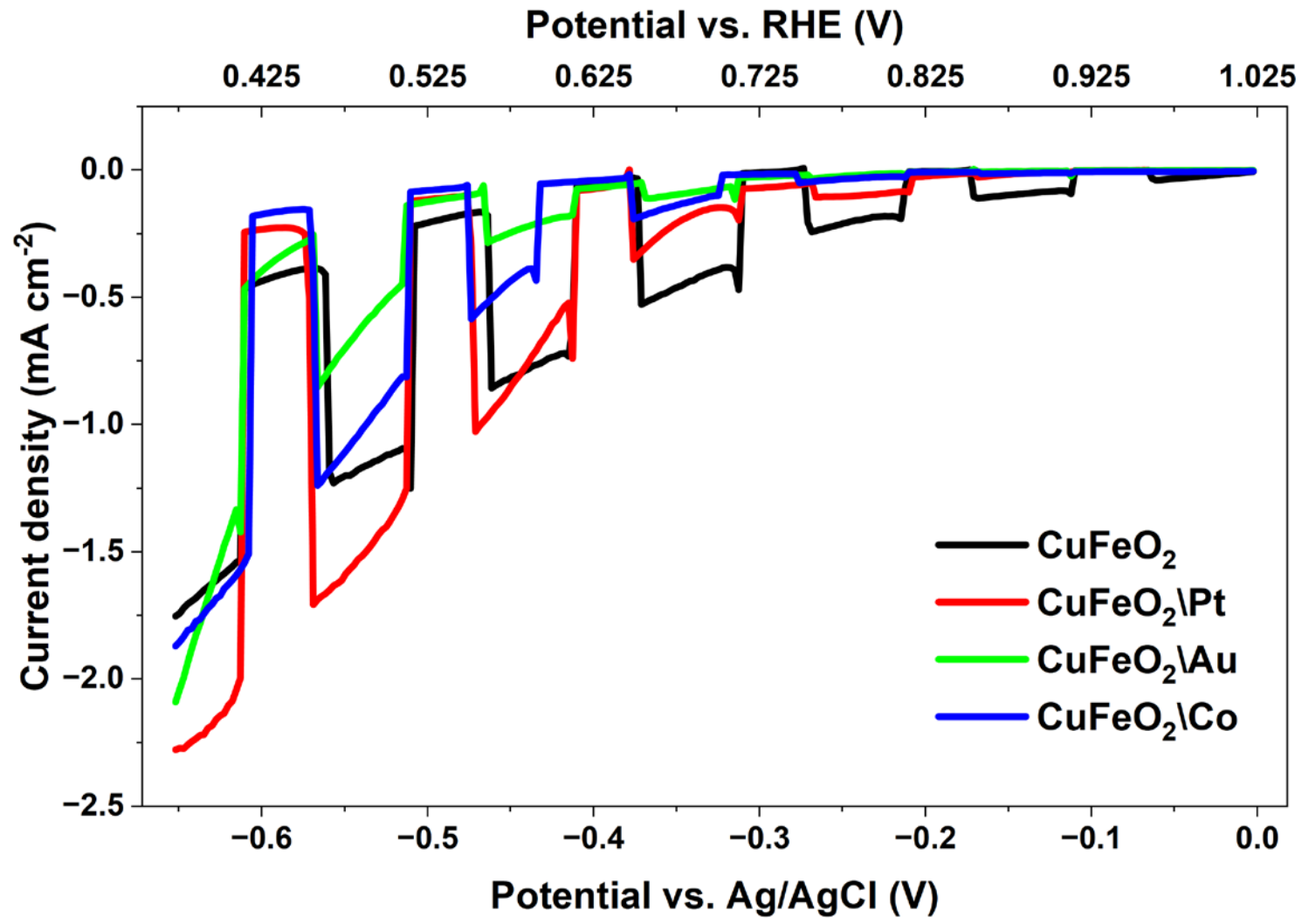


**Figure S7: Linear sweep voltammetry (LSV) curves of bare and modified $CuFeO_2$ photocathodes (Pt, Au, Co) recorded in $N_2$-purged 1 M NaOH. Measurements were performed under AM 1.5G illumination (100 mW cm$^{-2}$).**

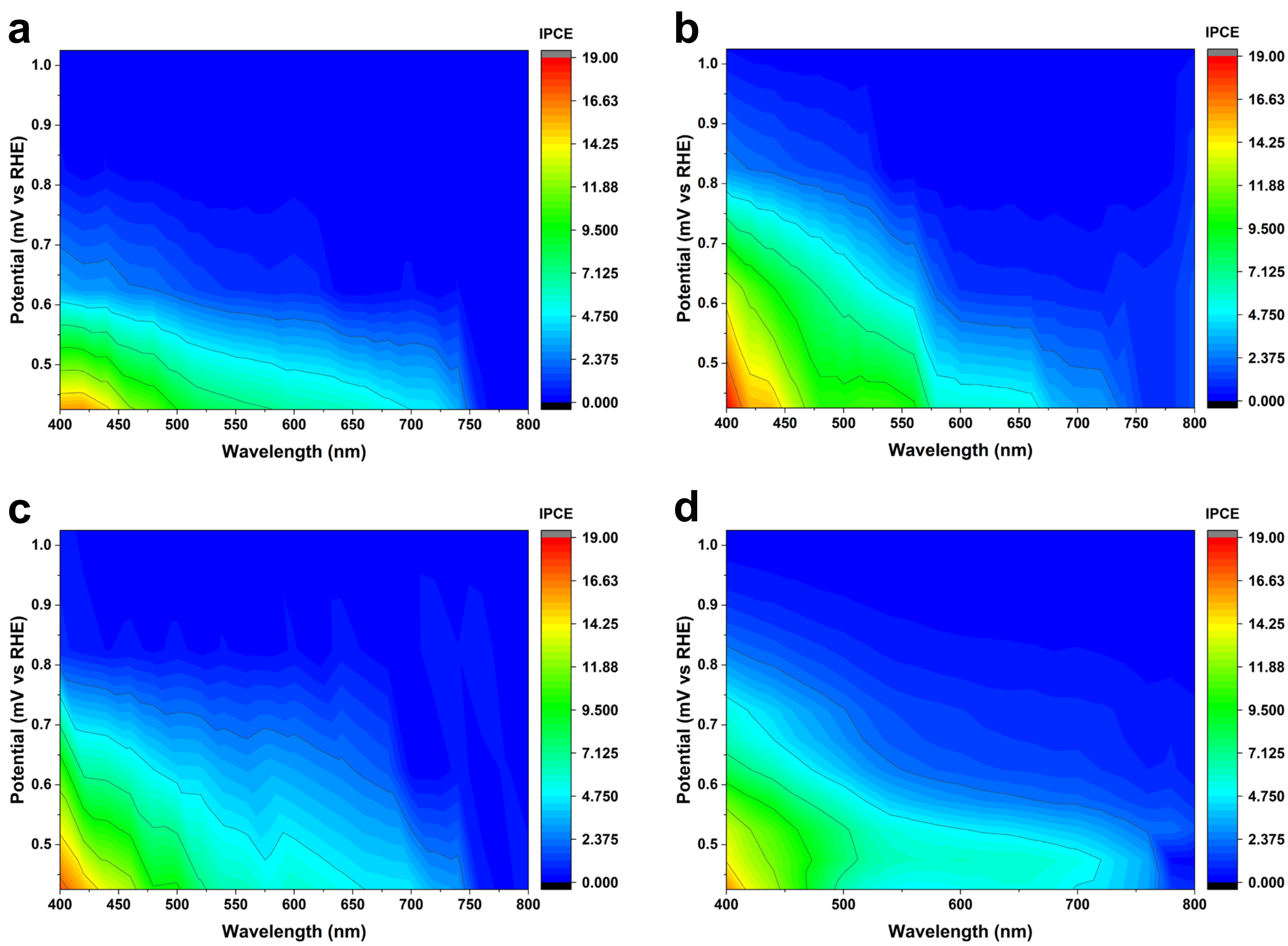


**Figure S8: Incident photon-to-current efficiency (IPCE) for (a) bare $CuFeO_2$, (b) $CuFeO_2$/Pt, (c) $CuFeO_2$/Au and (d) $CuFeO_2$/Co measured in 1.0 M NaOH with 100 mM $KNO_3$, highlighting the enhanced photon-to-electron conversion efficiency with Au and Pt modifications.**

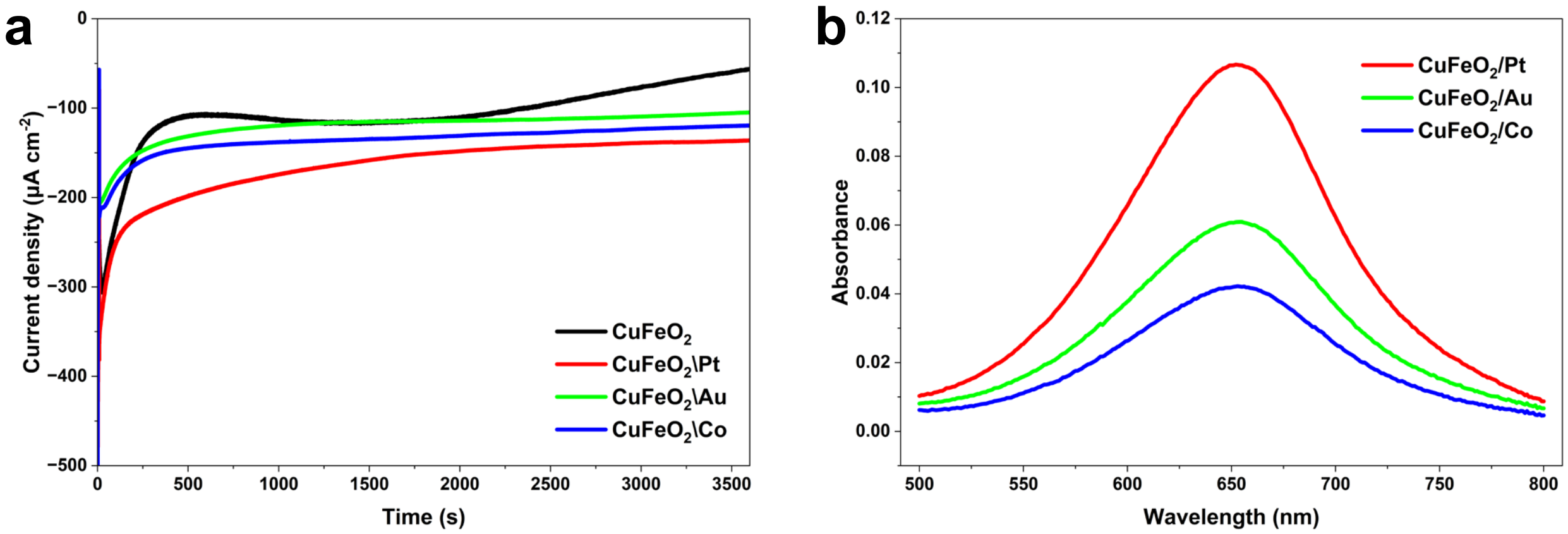


**Figure S9: (a) Chronoamperometry performed at 0.63 V vs. RHE for 1 hour under AM 1.5G illumination. Comparison of the stability of bare and modified $CuFeO_2$ photocathodes. (b) $NH_3$ quantification: UVvis absorbance spectra obtained for $CuFeO_2$ modified with Pt, Au and Co.**

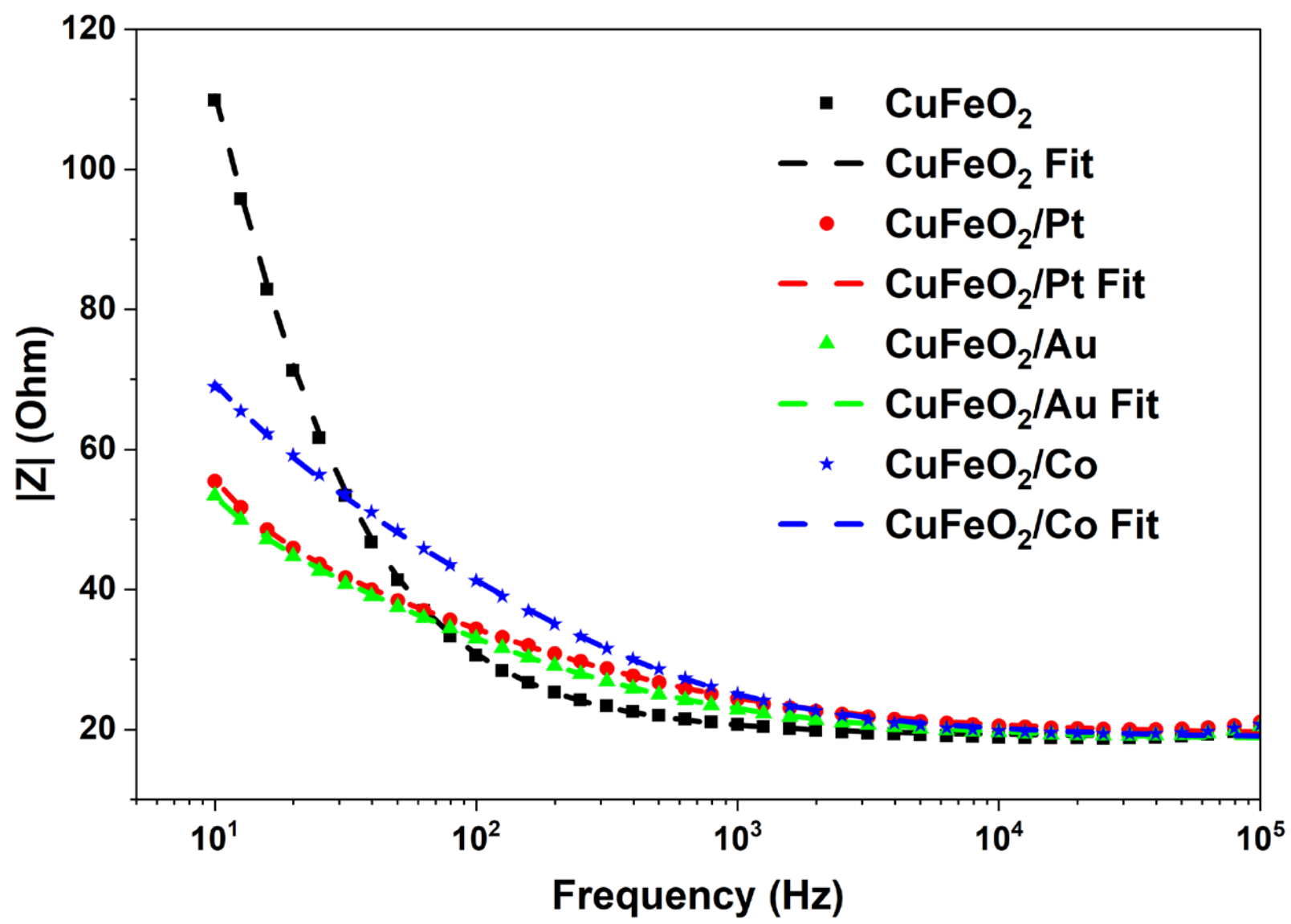


**Figure S10: Bode magnitude plots showing impedance modulus |Z| as a function of frequency for $CuFeO_2$ and modified photocathodes. The plots provide a frequency-domain representation of the EIS data shown in Figure 4 and show a lower low-frequency impedance response for the modified photocathodes compared with pristine $CuFeO_2$.**

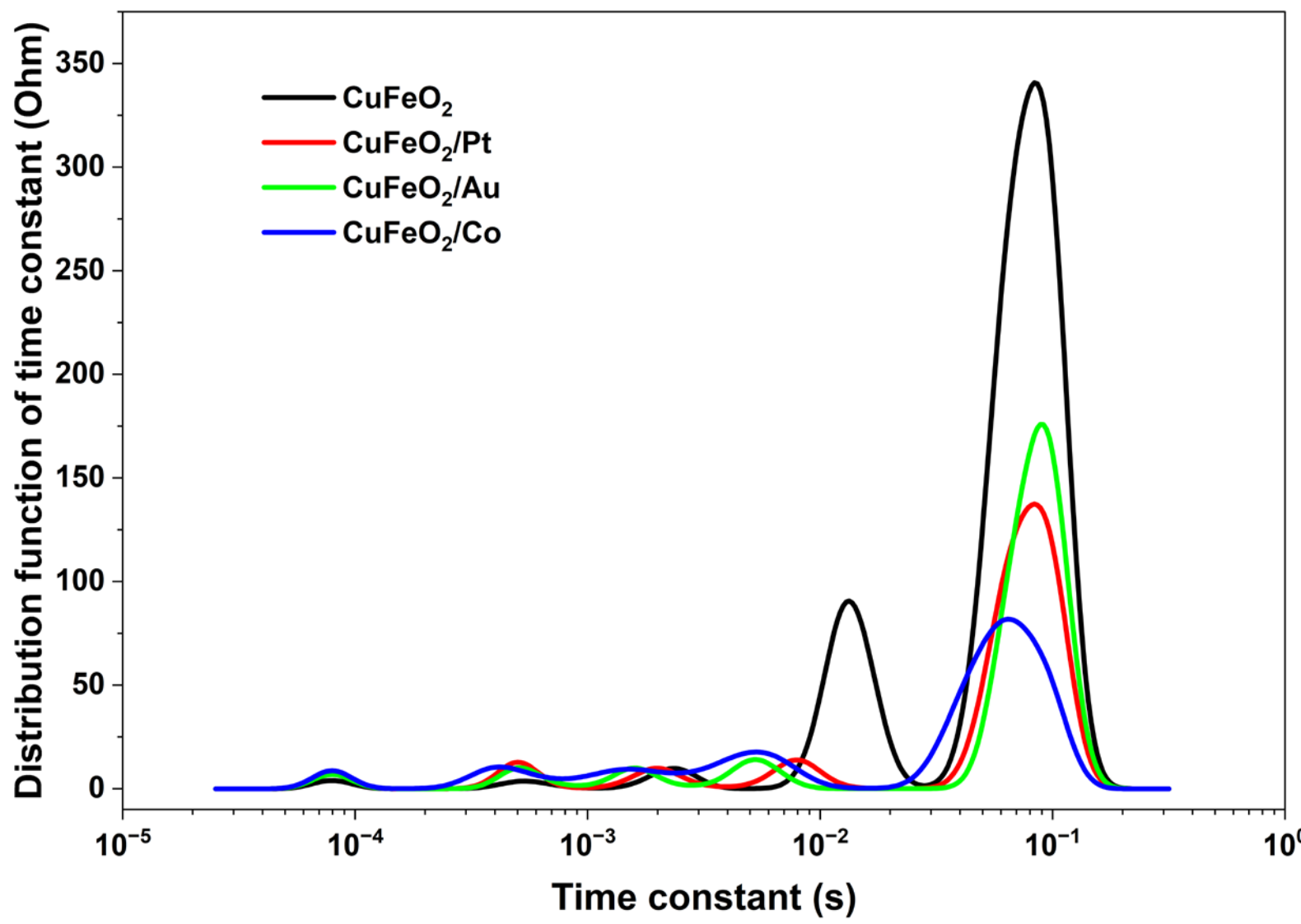


**Figure S11: Distribution of relaxation times (DRT) analysis of pristine and modified $CuFeO_2$ photocathodes under illuminated p-$NO_3$RR conditions. The DRT spectra visualize relaxation-time contributions within the measured frequency range and support the use of a two-time-constant equivalent-circuit model. Pristine $CuFeO_2$ shows two pronounced relaxation features, whereas the modified samples exhibit one dominant relaxation process with minor high-frequency contributions.**

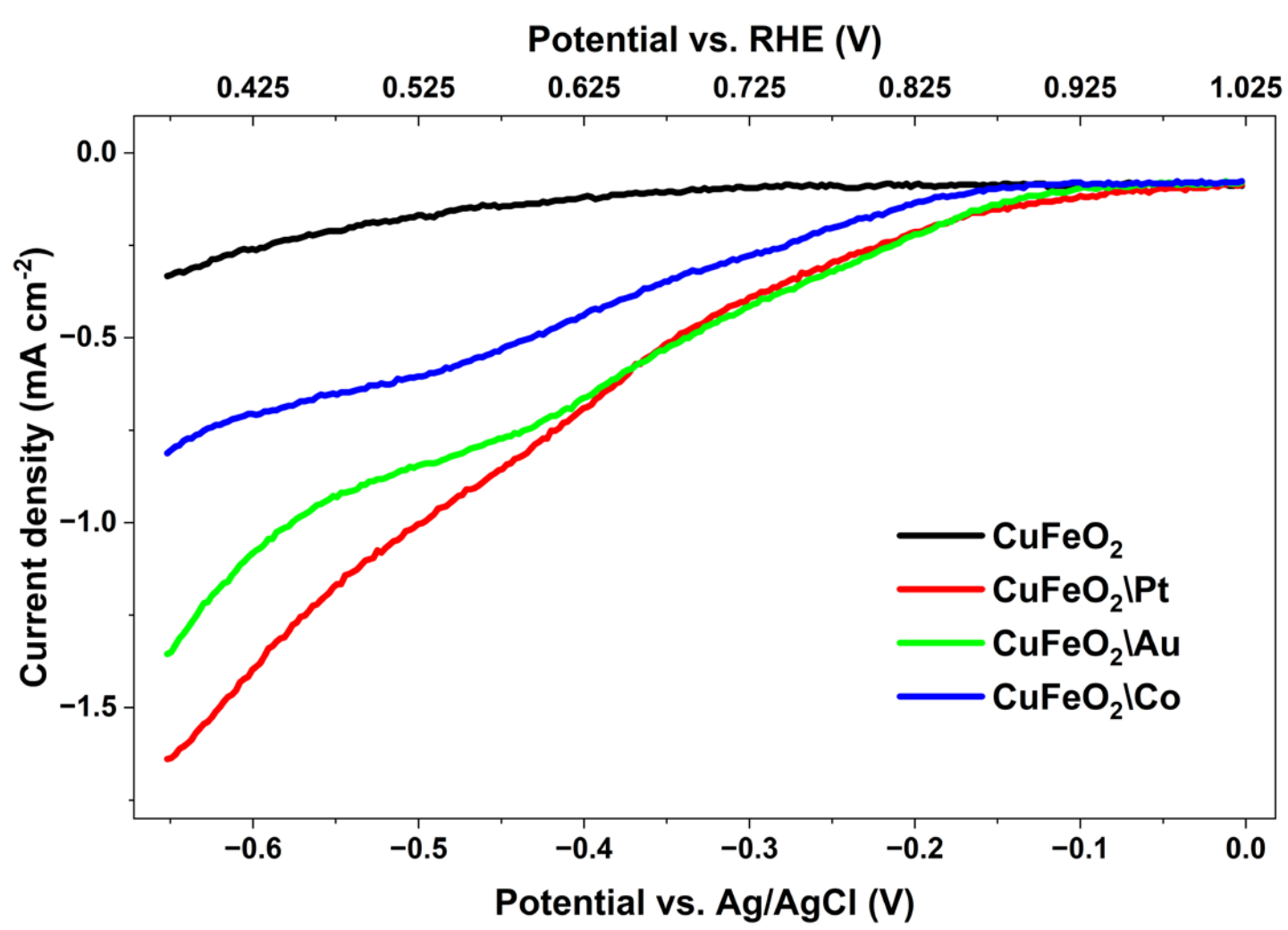


**Figure S12: Linear sweep voltammetry (LSV) curves of bare and modified $CuFeO_2$ photocathodes recorded after 1-hour PEC nitrate reduction. The measurements were performed in 100 mM $KNO_3$ / 1.0 M NaOH under 1-sun illumination. The data show complete deactivation of the pristine $CuFeO_2$ electrode and preserved activity for all surface-modified samples, confirming the stabilizing effect of Pt, Au, and Co cocatalysts.**

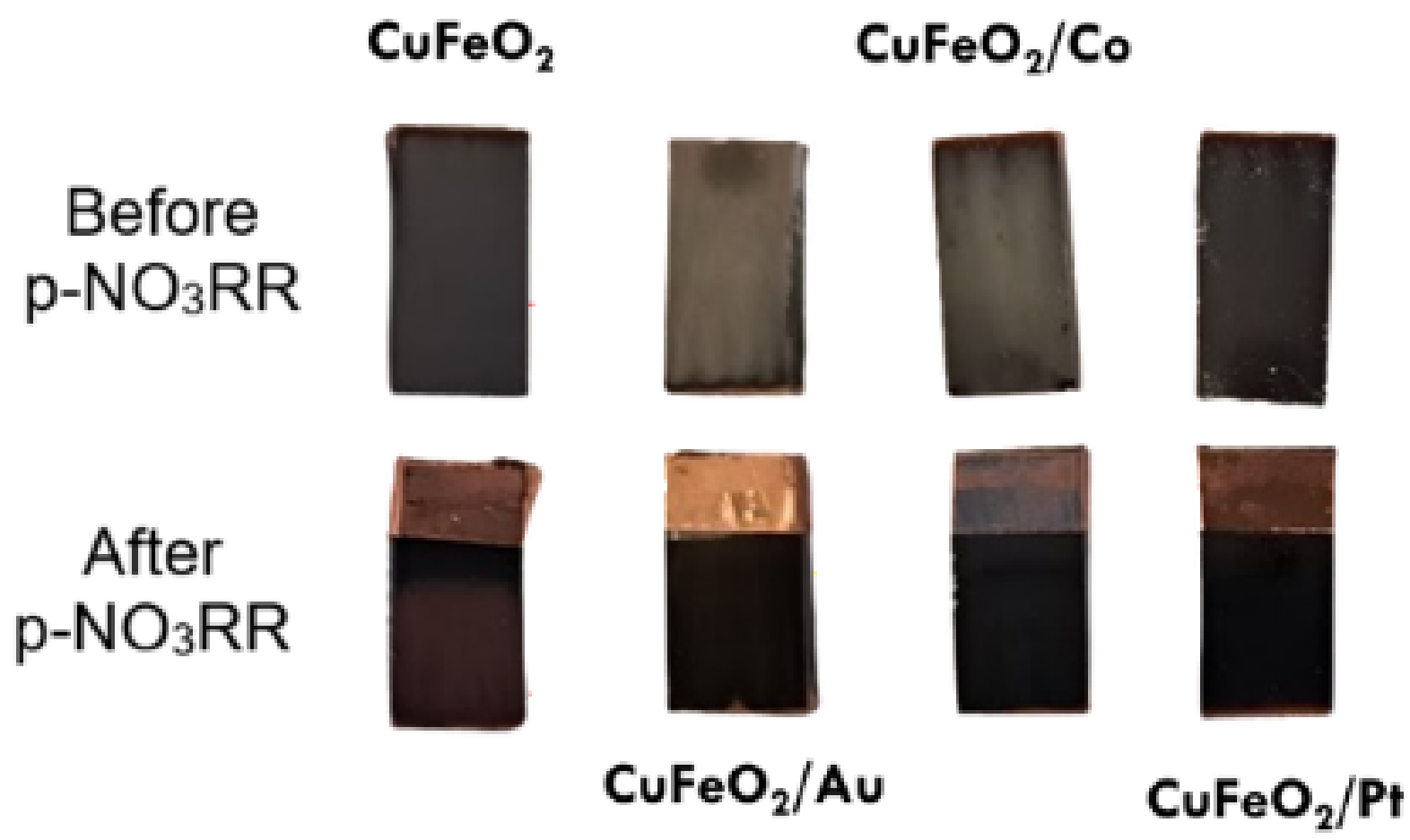


**Figure S13: Visual appearance of $CuFeO_2$ photocathodes before and after PEC nitrate reduction, illustrating the degradation of bare $CuFeO_2$ and the stability of modified electrodes.**

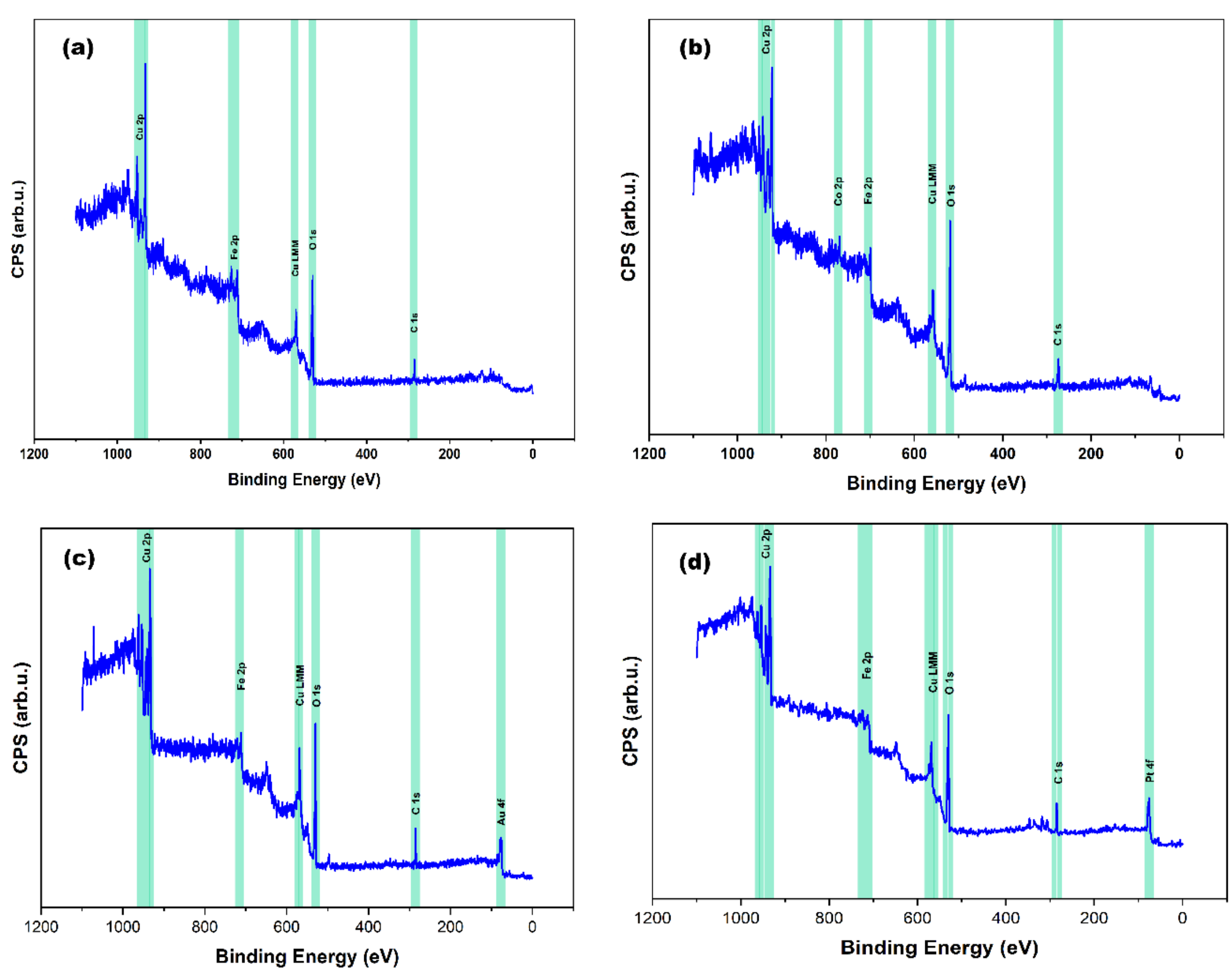


**Figure S14: XPS survey spectrum of the pristine and modified $CuFeO_2$ photocathodes. (a) bare $CuFeO_2$, (b) $CuFeO_2$/Co, (c) $CuFeO_2$/Au and (d) $CuFeO_2$/Pt.**

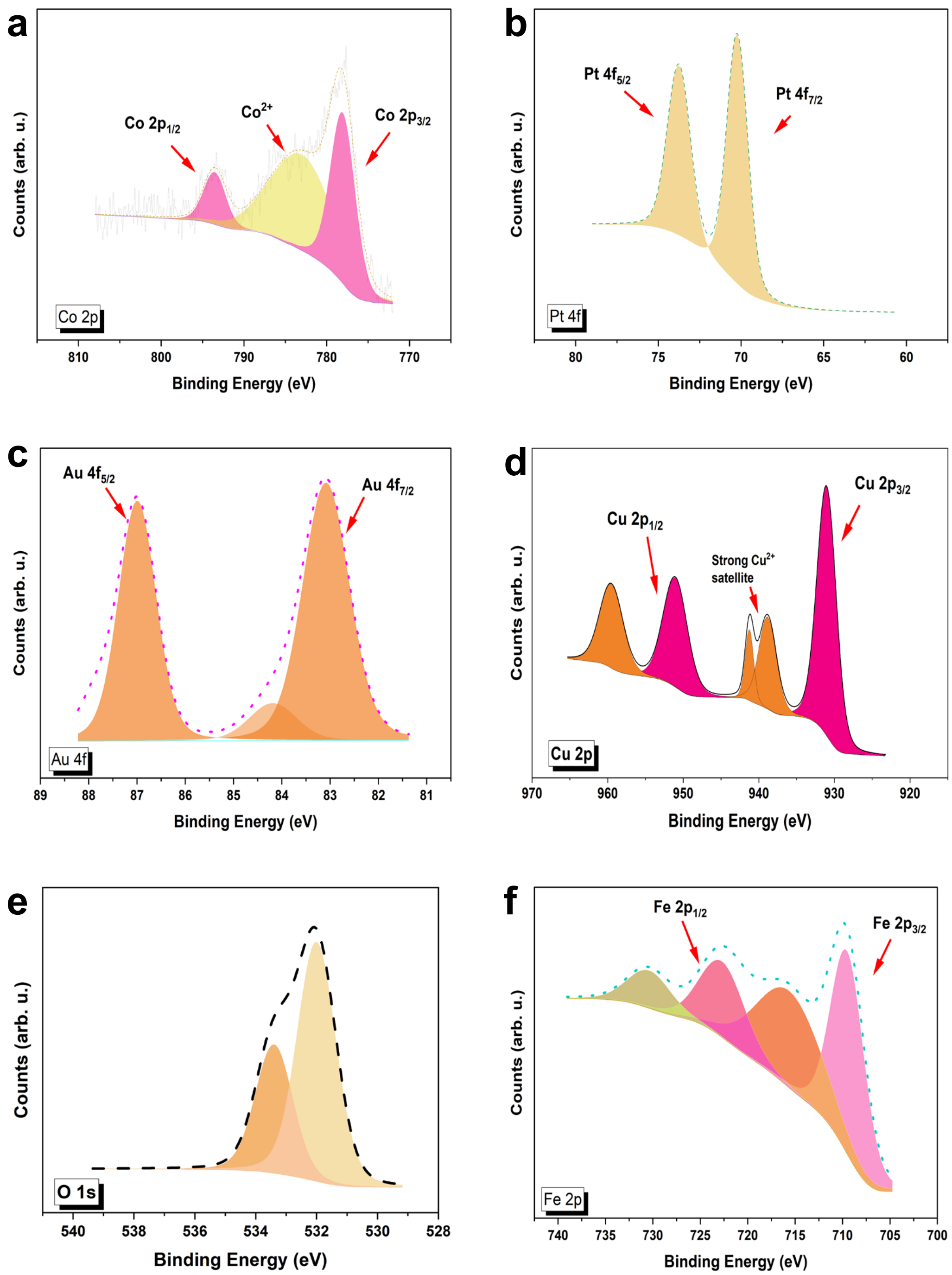


**Figure S15: High-resolution XPS spectra of modified $CuFeO_2$ surfaces recorded after p-$NO_3$RR. (a) Co 2p region for $CuFeO_2$/Co, (b) Pt 4f region for $CuFeO_2$/Pt, (c) Au 4f region for $CuFeO_2$/Au, (d) Cu 2p, (e) O 1s, and (f) Fe 2p regions obtained for $CuFeO_2$/Pt.**

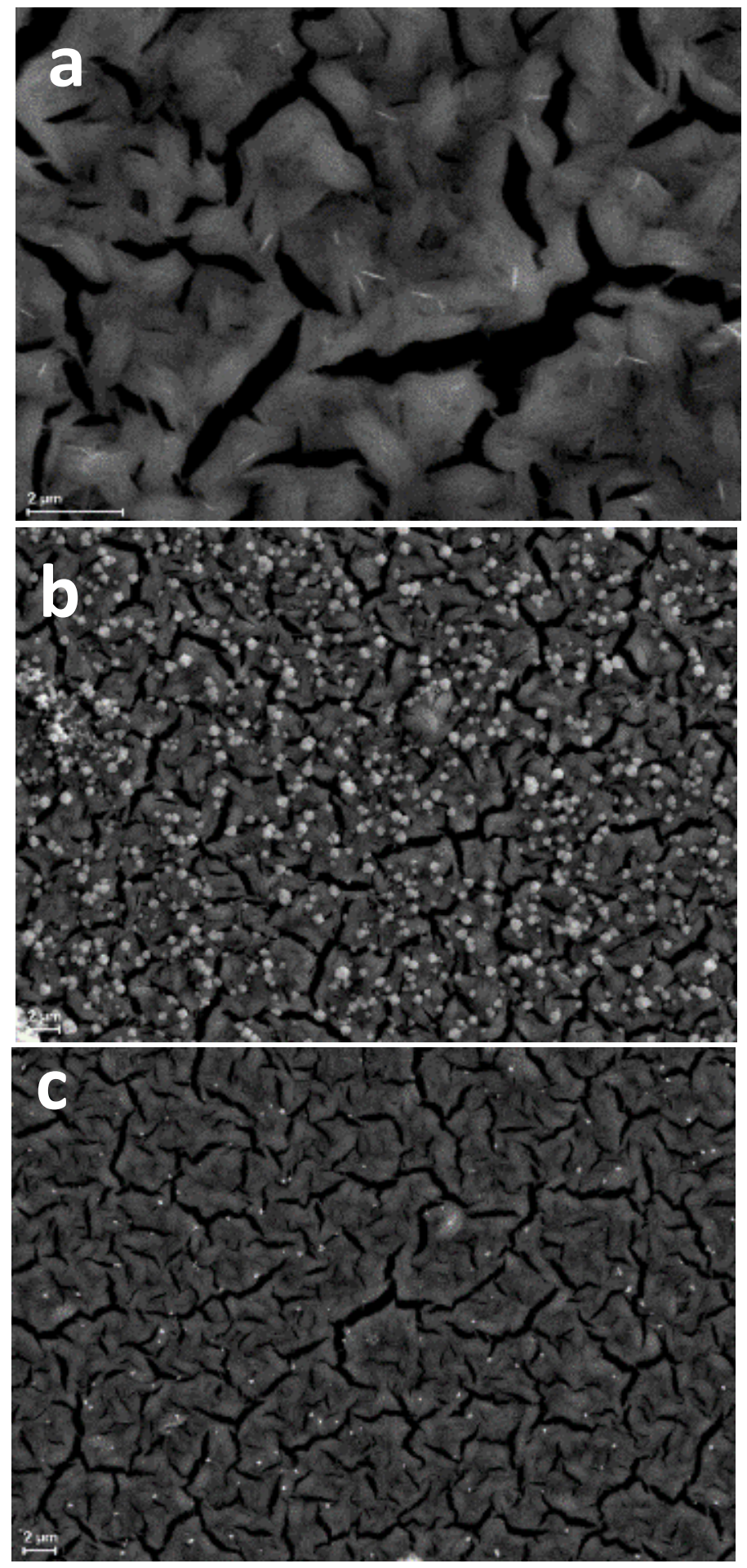

**Figure S16: SEM images of: (a) Pt-modified $CuFeO_2$ after PEC nitrate reduction, demonstrating preserved morphology. (b) Co-modified $CuFeO_2$ after PEC nitrate reduction. (c) Au-modified $CuFeO_2$, showing partial detachment of Au nano-particles after PEC nitrate reduction.**

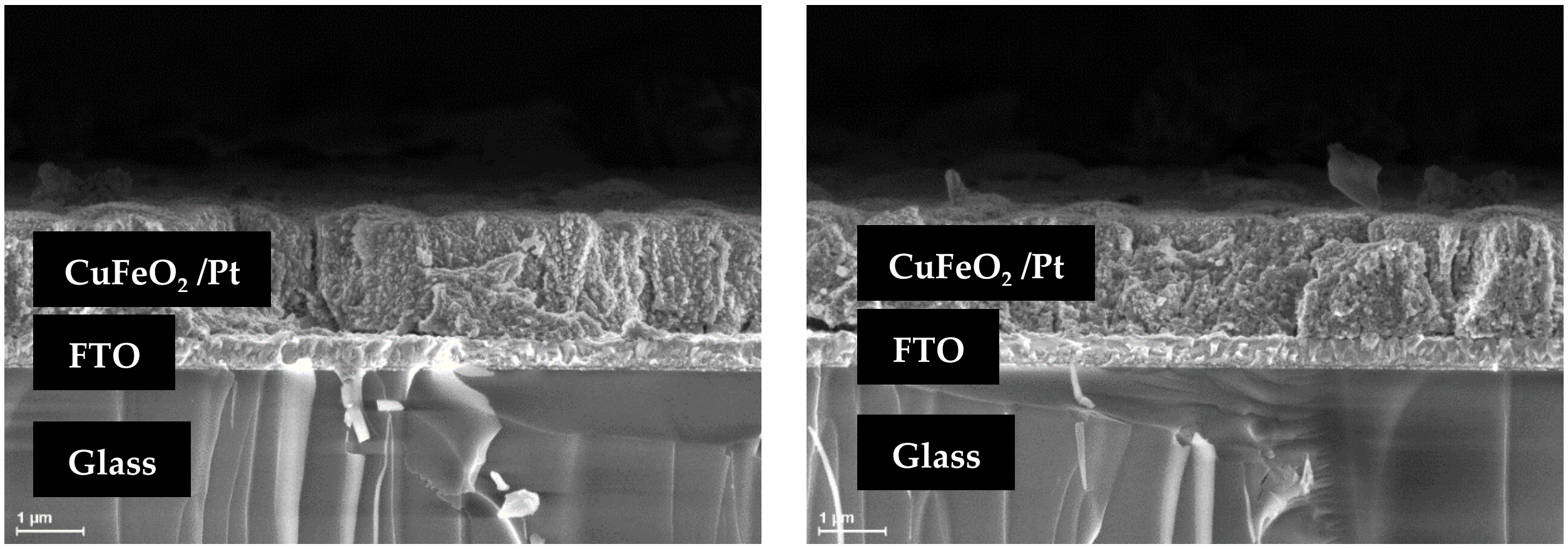


**Figure S17: Cross-sectional SEM images of $CuFeO_2$/Pt photocathodes before and after 1-hour PEC nitrate reduction under AM 1.5G illumination. The layered structure, film thickness, and interface quality remain unchanged after operation, confirming bulk morphological stability. Similar results were observed for bare and other modified electrodes.**

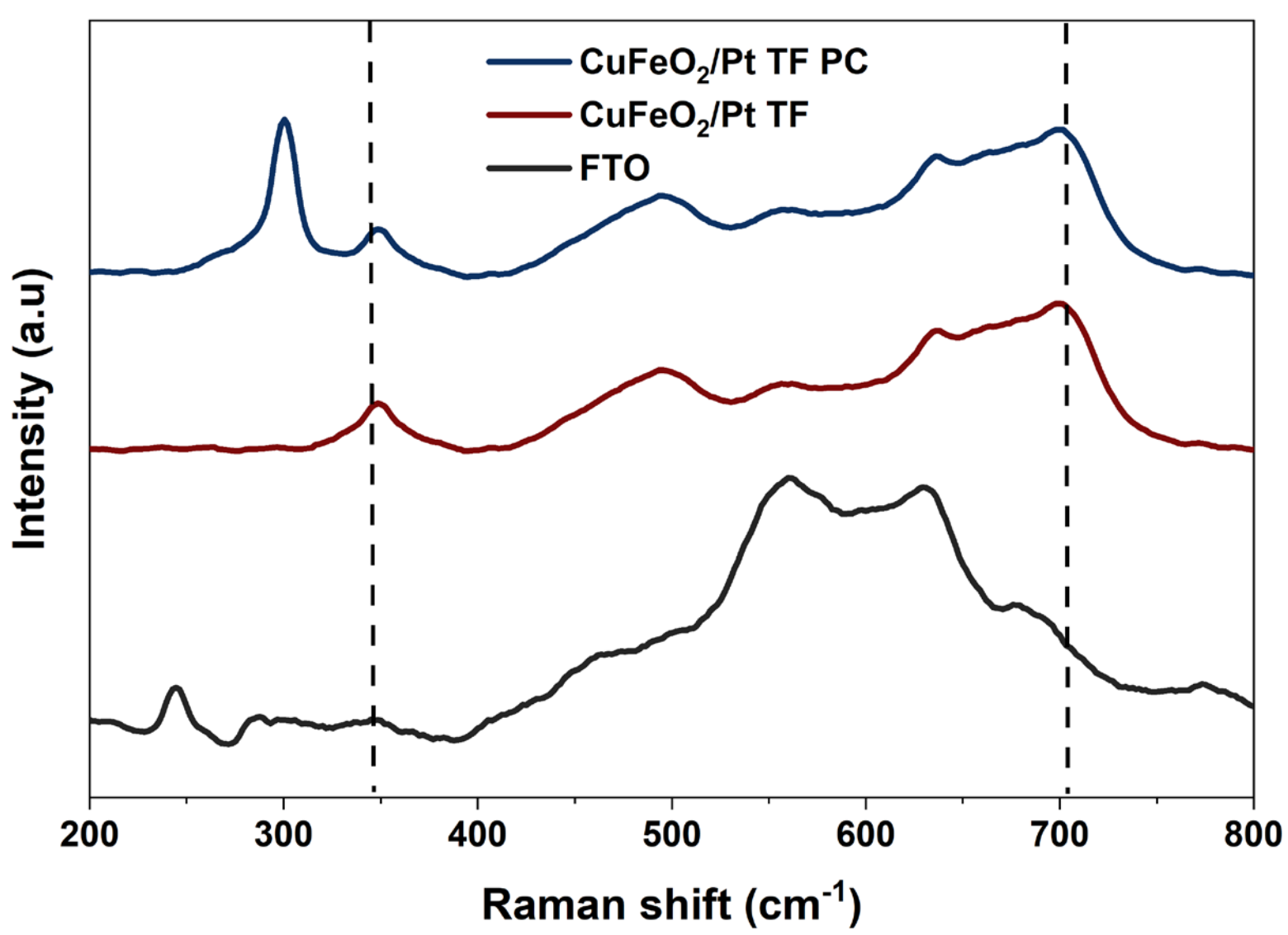


**Figure S18: Raman spectra of FTO substrate, $CuFeO_2$/Pt thin film (TF), and $CuFeO_2$/Pt thin film after post-catalysis (TF PC). Vertical dashed lines mark the Eg (~342 $cm^{-1}$, Cu–O stretching) and $A_1g$ (~710 $cm^{-1}$, O–Cu–O bending) modes characteristic of the 3R-$CuFeO_2$ phase.**

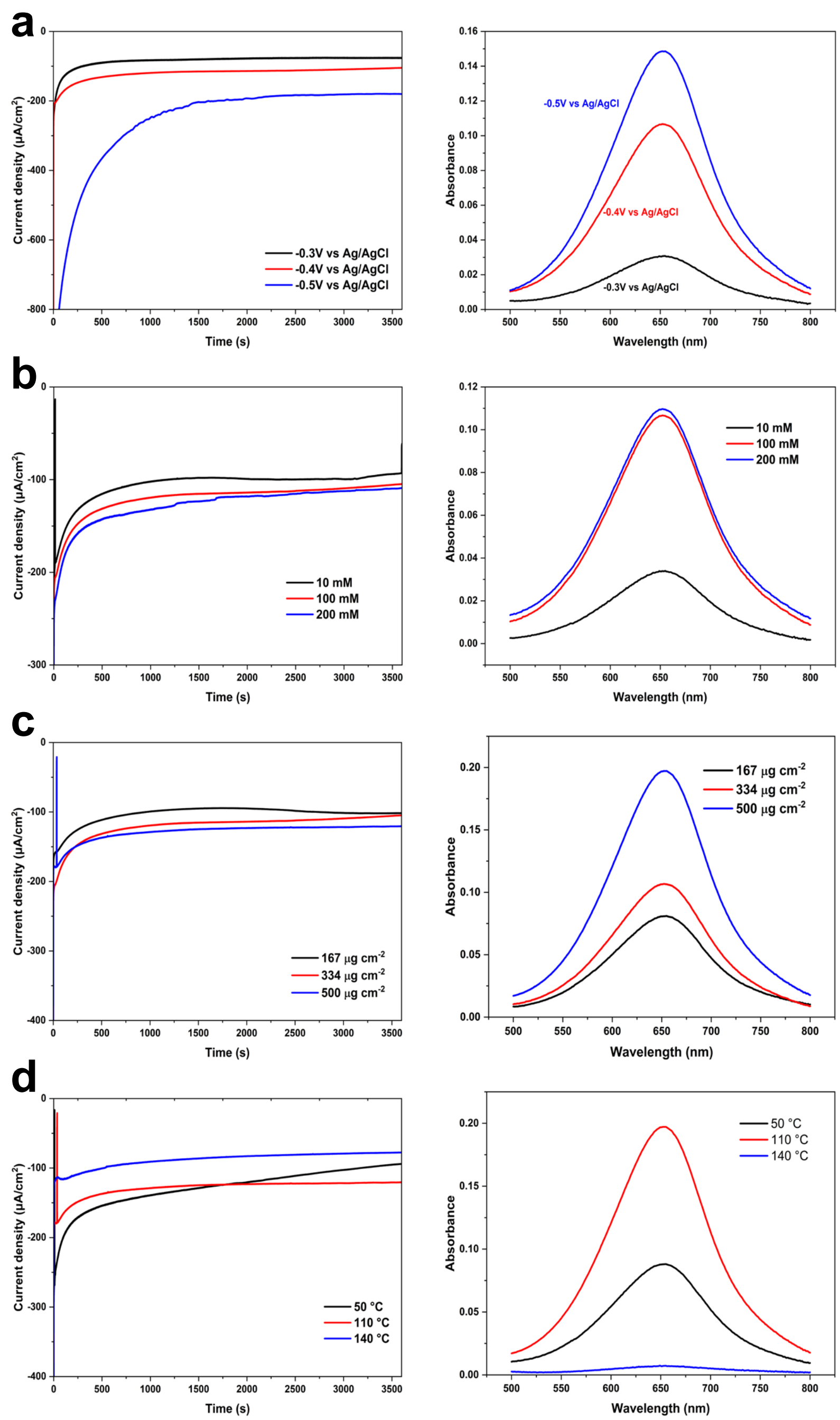


**Figure S19: Representative paired chronoamperometry (CA) traces and corresponding UV–Vis absorbance spectra used for $NH_3$ quantification and method validation. Each panel contains a matched CA trace (left) and the UV–Vis spectra (right) obtained from electrolyte aliquots taken during or immediately after the CA experiment. (a) Potential dependence: CA traces and paired absorbance spectra recorded at the potentials used for FE and rate analysis. (b) $NO_3^-$ concentration dependence: CA and absorbance pairs for representative concentrations (10, 100, 200 mM) showing saturation of FE and production rate near 100 mM. (c) Pt loading dependence: CA and absorbance pairs for Pt coverages (167, 337, 500 μg $cm^{-2}$) demonstrating increased photocurrent with loading and the associated $NH_3$ yields. (d) Pt deposition temperature: CA and absorbance pairs for electrodes prepared at 50, 110 and 140 °C, supporting the identification of 110 °C as the optimal deposition temperature.**

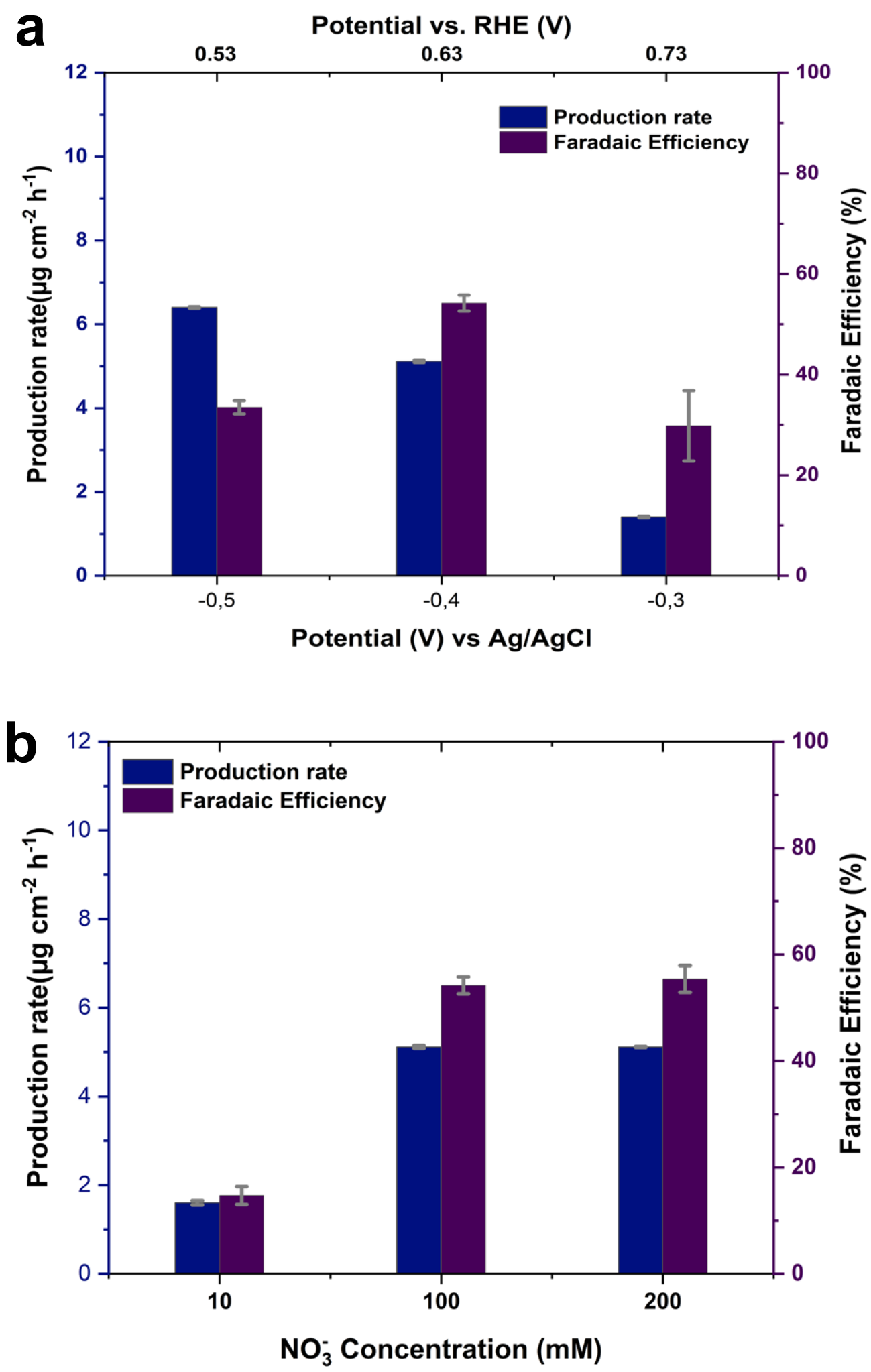


**Figure S20: Comparative ammonia production and Faradaic efficiency for $CuFeO_2$/Pt photocathodes. (a) Potential dependence of $NH_3$ Faradaic efficiency and production rate. (b) $NO_3^-$ concentration dependence of $NH_3$ Faradaic efficiency and production rate for optimized $CuFeO_2$/Pt (representative concentrations: 10, 100, 200 mM).**

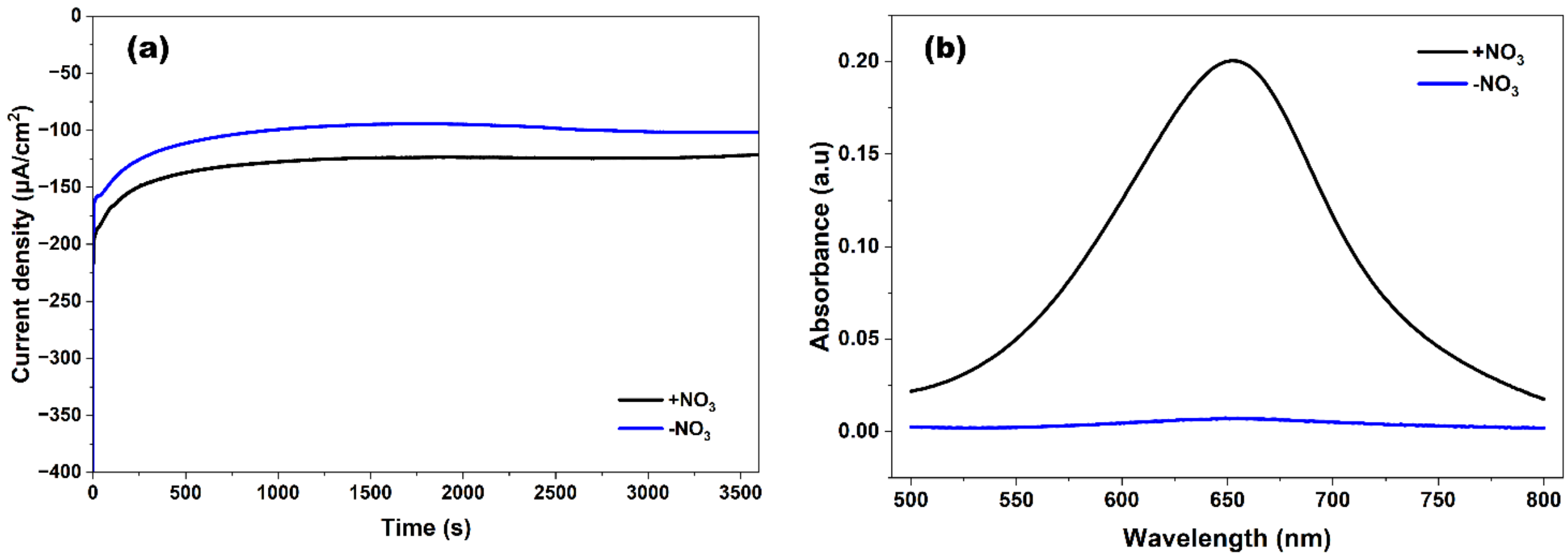


**Figure S 21: (a) Chronoamperometry (CA) of optimized $CuFeO_2$ photocathodes with and without nitrate in 1 M NaOH. A photocurrent is observed in nitrate-free electrolyte due to HER, while a higher photocurrent in the presence of nitrate. (b) UV-Vis absorbance spectra of indophenol assay solutions after 1 h PEC operation. A clear peak at 655 nm is detected only in nitrate-containing electrolyte, confirming ammonia formation from $NO_3$RR.**

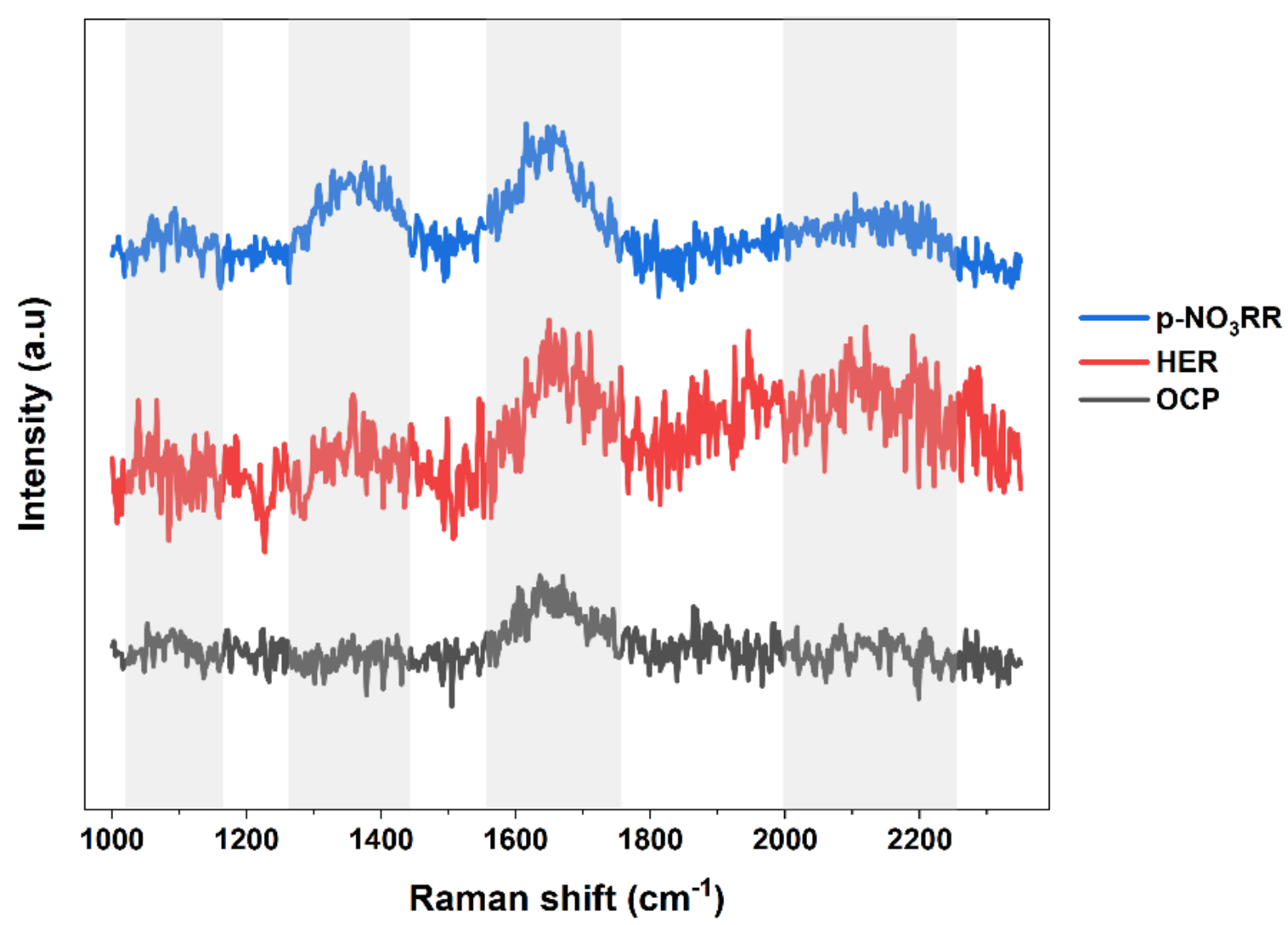


**Figure S22. Operando Raman spectrophotoelectrochemistry of $CuFeO_2$/Pt photocathodes under different electrochemical states. (a) Open-circuit potential (OCP) in HER environment showing the interfacial water bending vibration at 1647 $cm^{-1}$ (b) HER bias in 1 M NaOH revealing the Pt–H stretching vibration at 2100 $cm^{-1}$, confirming chemisorbed atomic hydrogen. (c) $NO_3$RR condition (1 M NaOH + 0.1 M $KNO_3$) showing nitrogenous intermediates at 1050 and 1364 $cm^{-1}$ alongside the Pt–H band, directly evidencing the tandem $H_{(ads)}$-mediated mechanism.**

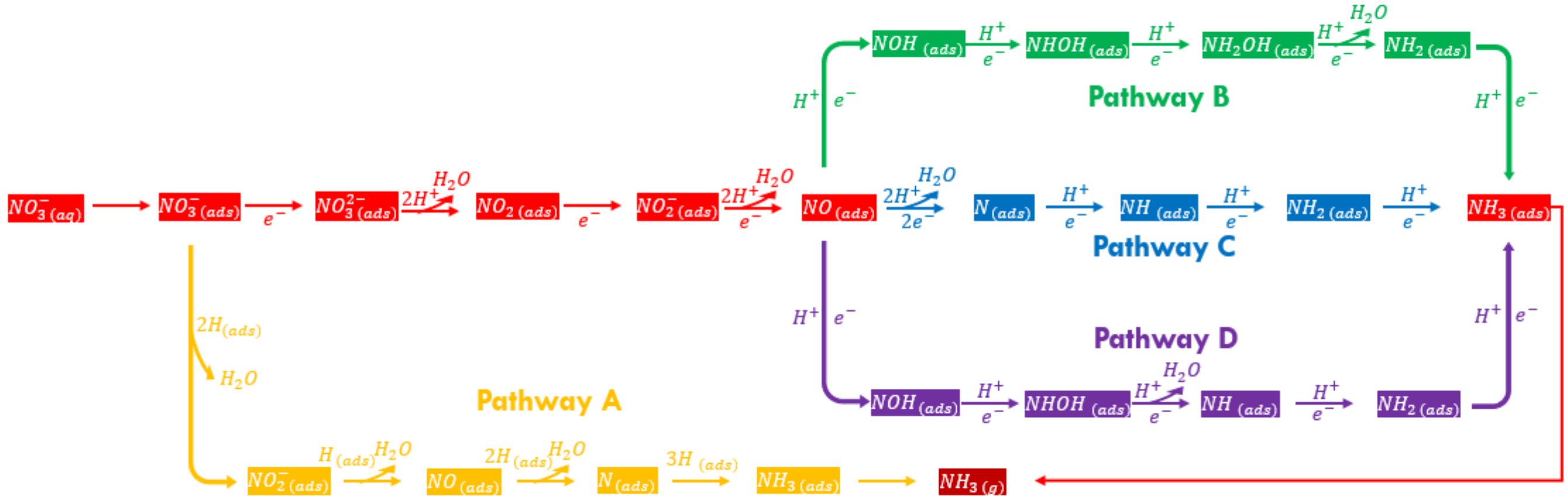


**Figure S23: Full $NO_3^- \rightarrow NH_3$ reaction pathway over $CuFeO_2$/Pt including proposed elementary steps ($NO_3^- \rightarrow NO_2^- \rightarrow NO \rightarrow N \rightarrow NH \rightarrow NH_2 \rightarrow NH_3$), key adsorbates and their relative migration barriers, and the role of Pt in lowering $H_{(ads)}$ migration barriers while Cu–Fe sites stabilize nitrogenous intermediates.**

**Table S1. Equivalent-circuit fitting parameters obtained from EIS spectra of pristine and modified $CuFeO_2$ photocathodes at 0.63 V vs RHE in 100 mM $KNO_3$ / 1.0 M NaOH under 1 sun illumination.**

| Sample | $R_0$ (Ω) | $R_1$ (Ω) | $R_2$ (Ω) | $\alpha_1$(CPE$_1$ exponent) | $\alpha_2$(CPE$_2$ exponent | $Q_1$ ($Fs^{\alpha-1}$) | $Q_2$ ($Fs^{\alpha-1}$) | $R^2$ (fit quality) |
|---|---|---|---|---|---|---|---|---|
| $CuFeO_2$ | 19.0 | 56.1 | 1 MΩ | 1.00 | 0.67 | $3.47\times10^{-4}$ | $9.64\times10^{-4}$ | 0.9986 |
| $CuFeO_2$/Au | 18.5 | 34.6 | 217.7 | 0.55 | 0.98 | $1.23\times10^{-3}$ | $8.54\times10^{-4}$ | 0.9971 |
| $CuFeO_2$/Co | 18.6 | 16.7 | 650.6 | 0.72 | 0.47 | $4.16\times10^{-4}$ | $3.44\times10^{-3}$ | 0.9986 |
| $CuFeO_2$/Pt | 19.3 | 27.6 | 131.1 | 0.55 | 0.91 | $1.11\times10^{-3}$ | $9.23\times10^{-4}$ | 0.9978 |

**Table S2: Literature comparison of PEC $N_2$ reduction catalysts, summarizing catalyst composition, reported Faradaic efficiency for $NH_3$ (%), operating potential (V vs. RHE), electrolyte and pH.**

| Category | Catalyst | FE for $NH_3$ (%) | Potential V vs RHE | Electrolyte (pH) | Reference |
|---|---|---|---|---|---|
| PEC $N_2$ reduction | Black Phosphorus | 23.3 | -0.4 | 0.1 M HCl (1) | Adv. Funct. Mater. 2020, 30, 2002731 |
| | $MoSe_2$@g-$C_3N_4$ | 28.91 | -0.3 | 0.1 M KOH (13) | J. Mater. Chem. A, 2021, 9, 2742 |
| | CuO | 17 | 0.6 | 0.1 M KOH (13) | J. Mater. Chem. A, 2021, 9, 2742 |
| | $Cu_2O$ | 20 | 0.4 | 0.1 M $Na_2SO_4$ | ACS Energy Lett. 2020, 5, 1834 |

**Table S3: Literature comparison of PEC $NO_3^-$ reduction catalysts, summarizing catalyst composition, reported Faradaic efficiency for $NH_3$ (%) and NO2 (%), operating potential (V vs. RHE), electrolyte and pH, with the present $CuFeO_2$/Pt system highlighted for direct comparison (FE = 92% at 0.6 V vs. RHE in 1.0 M NaOH + 100 mM $NO_3^-$). A more general overview is provided in Ref.[7]**

| Category | Catalyst | FE for $NH_3$ (%) | FE for $NO_2$ (%) | Potential V vs RHE | Electrolyte (pH) | Reference |
|---|---|---|---|---|---|---|
| PEC $NO_3$ - reduction | $Cu_2O$ | 20 | 81 | 0.0 | 0.5 M $Na_2SO_4$(6) | Artificial Photosynthesis 1.2 (2025): 97-105. |
| | $Cu_2O$ | - | > 85 | 0.4 | 0.1 M $K_2SO_4$(7) | ACS Energy Letters 9 (2024): 1993-1999. |
| | O_SiNW/Au (Si nanowire + Au cocatalyst) | 85 | - | 0.1 | 0.5 M $K_2SO_4$(3.5) | Angew. Chem. Int. Ed. 61 (2022): e202204117 |
| | Cu–Si NWs (Cu-decorated black silicon) | 97 | - | -0.4 | 0.5 M $H_2SO_4$ (0.3) | Energy Environ. Sci. 17 (2024): 9233–9243 |
| | Co–$TiO_x$/Ag/$TiO_x$/CdS/$Cu_2ZnSnS_4$ | 98 | - | 0.0 | 0.1 M KOH (14) | Applied Catalysis B: Environment and Energy 348 (2024): 123836 |
| | $CuFeO_2$/Pt | 92 | 0 | 0.6 | 1 M NaOH (13.2) | This work |

**References**


1. EL Idrissi, M.; Pöttker-Menke, J.; Kong, Y.; Abd-Lefdil, M.; Atourki, L.; Mei, B. Design of Experiment guided Hydrothermal Synthesis of CuFeO2 Photocathodes for Photoelectrochemical Hydrogen Evolution. Mater. Adv. 2026. DOI: 10.1039/D6MA00088F.
2. Giner-Sanz, Juan José, et al. "Salicylate method for ammonia quantification in nitrogen electroreduction experiments: the correction of iron III interference." Journal of The Electrochemical Society 167.13 (2020): 134519.
3. Searle, Phillip L. "The Berthelot or indophenol reaction and its use in the analytical chemistry of nitrogen. A review." Analyst 109.5 (1984): 549-568.
4. Carvalho, A. P., L. A. Meireles, and F. Xavier Malcata. "Rapid spectrophotometric determination of nitrates and nitrites in marine aqueous culture media." Analusis 26.9 (1998): 347-351.
5. Polatides, C., and Georgios Kyriacou. "Electrochemical reduction of nitrate ion on various cathodes–reaction kinetics on bronze cathode." Journal of Applied Electrochemistry 35.5 (2005): 421-427.
6. Berg, Richard, and Erna Becker. "Ein neuer Nachweis von Hydroxylamin durch Bildung von Chinolinchinon-(5.8)-[8-oxy-chinolyl 5-imid]-(5), genannt „Indo-oxin"." Berichte der deutschen chemischen Gesellschaft (A and B Series) 73.3 (1940): 172-173.
7. Y. Fang, Y. Gao, M. Li, Y. Wen, B. Shan. "Advances in photoelectrochemical nitrate reduction for solar ammonia synthesis" Chem Catalysis 6, 101600, February 19, 2026. DOI: 10.1016/j.checat.2025.101600.
